# An automated low-cost monitoring station for suspended sediments and water level

**DOI:** 10.1016/j.ohx.2024.e00594

**Published:** 2024-10-18

**Authors:** Paulo V.R.M. Silva, Robert B. James, Kathryn L. Russell, Tim D. Fletcher, Maria F.S. Gisi, Oldrich Navratil, Frederic Cherqui, Etienne Cossart

**Affiliations:** a University of Lyon, INSA Lyon, DEEP, EA7429, 69621 Villeurbanne, France; b WERG, SAFES, The University of Melbourne, Burnley, VIC 3121, Australia; c University of Lyon, CNRS UMR 5600 Environnement Ville et Société, Lyon, France; d University of Lyon, Université Claude Bernard Lyon-1, F-69622 Villeurbanne, France

**Keywords:** Suspended solids concentration (SSC), Flow cell, Stream, Water level, Autosampler, Turbidity

## Abstract

The use of low-cost sensors, with open-source code, facilitates greater spatial resolution and flexibility of environmental monitoring, thus generating more information and overcoming limitations of traditional commercial sensors. Measurement of water turbidity using submerged sensors can be problematic in that rapid biofouling requires frequent site visits to remove, clean, calibrate and replace the sensor. We therefore designed an automated system using low-cost commercially-available sensors that pumps water from the stream, samples it for turbidity and purges remaining water, leaving the turbidity sensor dry between measurements, thus greatly reducing the biofouling problem and minimizing operation costs. Our station was able to estimate suspended sediment concentrations between 0 and 6 g/L with a root mean square error (RMSE) around 5 % of the total range, which meets typical research and operational study requirements. The results showed that the monitoring station is capable of monitoring water level and turbidity for long periods without the need of cleaning the turbidity sensor, due to its purge function. We demonstrated that spatially intense measurement of turbidity within catchments and drainage networks can be achieved at a relatively low cost, which allows a better understanding of the main sources of suspended sediments and their spatial and temporal variability.

## Graphical abstract

**Figure:**
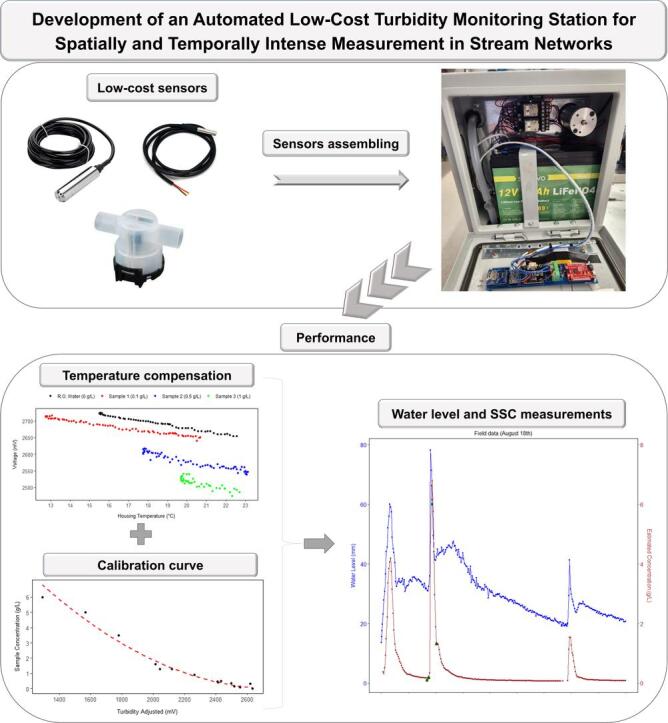

## 1 Specifications table

**Table:** 

Hardware name	Automated Low-Cost Mobile Turbidity Monitoring Station
Subject area	• Engineering and materials science • Environmental, planetary and agricultural sciences • Educational tools and open source alternatives to existing infrastructure
Hardware type	• Field measurements and sensors
Closest commercial analog	GUIDED WAVE™ Turbidity Process Flow Cell *https://www.process-insights.com/products-3/products-industrial/nir-uv*–*vis-process-and-lab-analyzers/flow-cells-probes/turbidity-process-flow-cell/*
Open source license	CC BY-SA 4.0
Cost of hardware	AUD$ 870.05
Source file repository	*https://osf.io/cxbpf/?view_only=378951f7e25949189b9773dd6cacd628*

## 2 Hardware in context

Soil erosion, often triggered by human activities such as intensive agriculture, mining, construction and deforestation, results in the mobilization of sediments which, consequently, increase water turbidity and introduce suspended particles into water bodies [1]. These particles, transported by water, can negatively affect water quality, compromising aquatic life, water security and human health [2], [3]. Turbidity levels (a visual indicator of sediment concentration in water) and total suspended solids (TSS) (a gravimetric measure of sediment concentration in water) are therefore essential to assess temporal and spatial variability in water quality, being important indicators of erosion in a watershed [4].

Several methods are used to estimate suspended sediment concentrations (SSC) [5]. The first involves direct measurements by sampling the water at regular or irregular intervals at a representative point and determining total suspended solids (TSS) by gravimetric analysis in a laboratory. This method is recognized for its reliability; however, it has some disadvantages, such as its discontinuity, and substantial costs, both financially and in time [6].

The other method consists of indirectly measuring SSC, by using multi- or hyperspectral images or turbidity probes. Multi- or hyperspectral images can be obtained by satellites, terrestrial devices or UAVs, and their reflectance values processed to estimate SSC. Although there are some studies that use this method to estimate SSC, it has several limitations, such as image resolution, riverbed and water surface reflection, poor spatial resolution, high cost and restrictions related to weather conditions [7], [8], [9]. These limitations render this method less suitable for long-term monitoring of SSC, especially in small streams [6]. Nowadays, it is common to use turbidity sensors to continuously measure SSC at high temporal resolution, where a strong relationship between turbidity and SSC can be established [6], [10].

Commercial turbidity sensors prioritise accuracy, reliability and usability in environmental measurements, and are currently widely used in field measurements. However, their high cost can be a limiting factor for studies that require continuous measurements at multiple points of interest in streams [11]. To address this limitation, low-cost turbidity sensors have been developed and documented in peer-reviewed journals by many authors, using different approaches and technologies.

Gillett and Marchiori (2019) [12] and Trevathan et al. (2020) [13] tested commercially available appliance turbidity sensors, such as those used in washing machines, making some adaptations to waterproof the turbidity sensor and to improve its performance. Some other authors have developed alternative strategies by using different wavelength light sources, such as near infrared, or adding more than 1 LED, thus having distinct wavelengths to estimate turbidity [11], [14]. Others have used more than one photodetector at different angles to perform more accurate measurements for different monitoring scenarios [6], [15], [16]. Additionally, some authors have implemented combined reading systems, that is, with LED off and on, in order to compensate for the effects of sunlight [11], [17], [18]. However, even with all these improvements made by several authors, problems related to temperature, fouling and ambient light continue to be major challenges to be overcome in *in-situ* monitoring.

To minimize and mitigate these effects, we developed an automatic low-cost turbidity monitoring station that functions as a flow cell, where water samples are pumped through the turbidity sensor for measurements. To our knowledge, our mobile turbidity monitoring station is the first low-cost flow-cell type system tested *in-situ* over a long period of time (May-November 2023). With an innovative design, our station is capable of monitoring water temperature, turbidity, suspended solids concentration, and water level in diverse environments, such as lakes, rivers, and stormwater pipes.

Traditional calibration methods involve initially calibrating the turbidity sensor using formazin solutions or costly polymer calibration standards. Subsequently, the sensor is calibrated with field-collected samples of known concentrations to establish a relationship between measured turbidity (NTU or FNU) and SSC (g/L). In contrast, our calibration approach utilizes sediment samples from the study site to establish a relationship between turbidity measurements (mV) and SSC (g/L). This method directly integrates field-specific conditions into the calibration process, offering a tailored solution that bypasses the need for standard formazin solutions or expensive polymer standards. Calibration and validation processes were performed using water samples with concentrations from 0 to 1 g/L of suspended bentonite (laboratory) and with water samples collected *in-situ* during the monitoring period with TSS concentrations ranging from 0 to 6 g/L.

## 3 Hardware description

To the best of our knowledge, all low-cost turbidity sensors developed and reported in the literature to measure *in-situ* turbidity and/or suspended solids concentration need to be submerged in water to make measurements. All such sensors suffer from problems such as fouling (e.g. algae growth), as they are always in contact with water, just like the high-cost commercial sensors available on the market.

With a novel design, we developed an automatic mobile suspended solids monitoring station capable of sampling water for reading its suspended solids concentration. Our station design reduces the effect of sedimentation, clogging and biofouling of the turbidity sensor, as it is positioned inside a cabinet and not within the water body. Additionally, positioning the turbidity sensor inside the cabinet avoids the influence of ambient light on its readings. The influence of ambient light is known as one of the main challenges of implementing low-cost optical turbidity sensors to monitor suspended sediments *in-situ* [12], [13]. The dark environment provided by the cabinet also minimizes the possibility of algal growth within the sensor housing.

Another common problem with optical turbidity sensors which use LED as a light source is that fluctuations in temperature affect the electrical output of the sensor [19]. Therefore, we adapted a commercial low-cost optical turbidity sensor (TST-10 [20]), which has an IR emitter LED source, by installing a temperature sensor (DS18B20 [21]) inside its housing, to record the sensor's internal temperatures and subsequently adjust the turbidity measurements and thus minimize the temperature effects.

Based on laboratory experiments, a 1 °C variation resulted in an average change of 10 mV in turbidity readings, which corresponds to an approximate variation of 0.8 % for the sample with the highest sediment concentration and 0.3 % for the sample with the lowest concentration. Therefore, for small temperature variations, the influence of these fluctuations on the overall accuracy of turbidity measurements is minimal.

Our monitoring station has a peristaltic pump comparable in flow rate and flow velocity with the autosampler Hach Sigma 900 Max pump, which facilitates comparison between samples collected with both systems.

In addition, our system also has a purge function, which eliminates all water from the sample collection system (tubes and sensor). Therefore, the turbidity sensor is not in contact with water during the intervals between turbidity measurements, which minimizes the likelihood of sediment sticking to the inner walls of the sensor housing and, additionally, prevents algae growth. This reduces the number of station maintenance visits and, consequently, the cost of operation during monitoring.

Our system also differs from all others present in the literature in that it reads dry turbidity, in other words, the turbidity of the air, just before the turbidity measurements of the water sample. This is a mechanism for checking the need for station maintenance, such as changing the batteries, and cleaning the turbidity sensor. For example, if air turbidity values decrease over time, this is an indication that the sensor needs cleaning. Additionally, if the turbidity values in dry and wet conditions are the same, this may indicate that the peristaltic pump is not activating due to battery depletion.

Our monitoring station has also a waterproof temperature sensor (DS18B20 [21]) to measure water temperature, which can provide valuable information about the monitored environment. Furthermore, it helps with post-processing of the data, as large differences between the temperature of the water and the sensor housing can cause fluctuations in its turbidity measurements.

The monitoring station has a water level sensor, that activates the peristatic pump to measure turbidity in situations where a defined minimum water elevation trigger is reached. Furthermore, water levels are recorded allowing estimation of flow rates, which, combined with continuous monitoring of the SSC, allows estimation of sediment loads and yields. In addition, the station is set to sleep mode between measurements, reducing power consumption and thus increasing autonomy of the monitoring site.

Finally, the station features a.txt file on the micro-SD card that allows the operator to change the station’s measurement settings, such as the trigger level to activate the pump, time between measurements, pump activation time (forward and reverse modes), number of turbidity measurements, and many other settings. Therefore, its open-source code allows these measurement characteristics to be easily changed, which facilitates adaptation to suit the desired monitoring situation.

## 4 Design files summary

**Table:** 

Design file name	File type	Open source license	Location of the file
Electronic circuit schematic.JPG	JPEG	CC-BY-4.0	Available with the article / https://osf.io/cxbpf/?view_only=378951f7e25949189b9773dd6cacd628
Portable_Turbidity_Station.ino	Arduino	CC-BY-4.0	https://osf.io/cxbpf/?view_only=378951f7e25949189b9773dd6cacd628
CFG_1.txt	text	CC-BY-4.0	Available with the article / https://osf.io/cxbpf/?view_only=378951f7e25949189b9773dd6cacd628
IP66 enclosure − cabinet_1.JPG	JPEG	CC-BY-4.0	Available with the article / https://osf.io/cxbpf/?view_only=378951f7e25949189b9773dd6cacd628
IP66 enclosure − cabinet_2.JPG	JPEG	CC-BY-4.0	Available with the article / https://osf.io/cxbpf/?view_only=378951f7e25949189b9773dd6cacd628
Turbidity sensor housing.JPG And	JPEG	CC-BY-4.0	Available with the article / https://osf.io/cxbpf/?view_only=378951f7e25949189b9773dd6cacd628
Assembling_1.JPG	JPEG	CC-BY-4.0	Available with the article / https://osf.io/cxbpf/?view_only=378951f7e25949189b9773dd6cacd628
Assembling_2.JPG	JPEG	CC-BY-4.0	Available with the article / https://osf.io/cxbpf/?view_only=378951f7e25949189b9773dd6cacd628
Assembling_3.JPG	JPEG	CC-BY-4.0	Available with the article / https://osf.io/cxbpf/?view_only=378951f7e25949189b9773dd6cacd628
Pump assembling.JPG	JPEG	CC-BY-4.0	Available with the article / https://osf.io/cxbpf/?view_only=378951f7e25949189b9773dd6cacd628
Assembling − Supplementary Information. pdf	PDF	CC-BY-4.0	Available with the article / https://osf.io/cxbpf/?view_only=378951f7e25949189b9773dd6cacd628
Recommendation to avoid bubbles − Supplementary Information. pdf	PDF	CC-BY-4.0	Available with the article / https://osf.io/cxbpf/?view_only=378951f7e25949189b9773dd6cacd628
Software user manual − Configuration file − Supplementary Information. pdf	PDF	CC-BY-4.0	Available with the article / https://osf.io/cxbpf/?view_only=378951f7e25949189b9773dd6cacd628

**Design file descriptions**

**Fig. 1:**
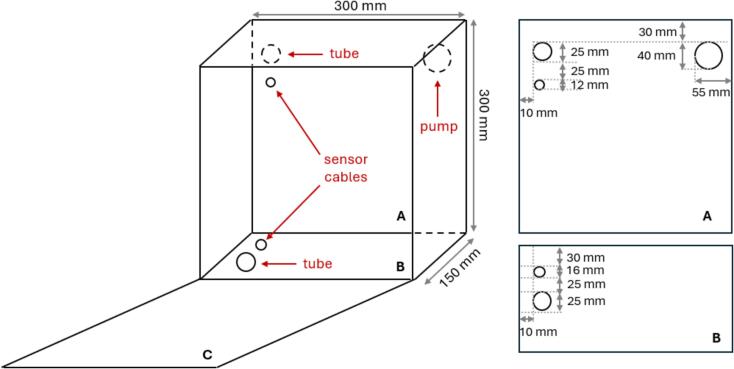
IP66 cabinet preparation. “A” is the back wall of the cabinet. Three holes were made on this wall to pass the pump, the temperature sensor cable and the outlet tube. “B” is the base wall of the cabinet. Two holes were made on this wall to pass the inlet tube and the water pressure sensor cable. “C” is the lid of the cabinet.

**Fig. 2:**
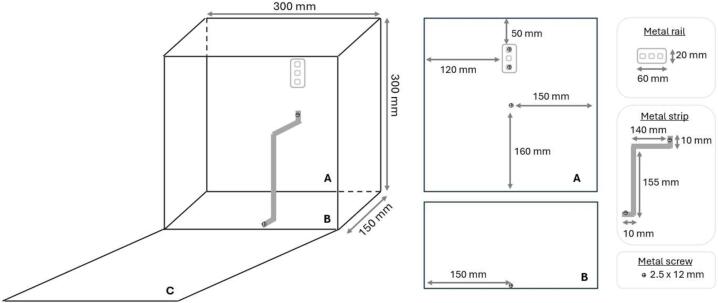
Details of the cabinet preparation. “A” is the back wall of the cabinet, where the metal rail and one end of the strip are fixed. “B” is the bottom wall of the cabinet, where the other end of the metal strip should be fixed.

**Fig. 3:**
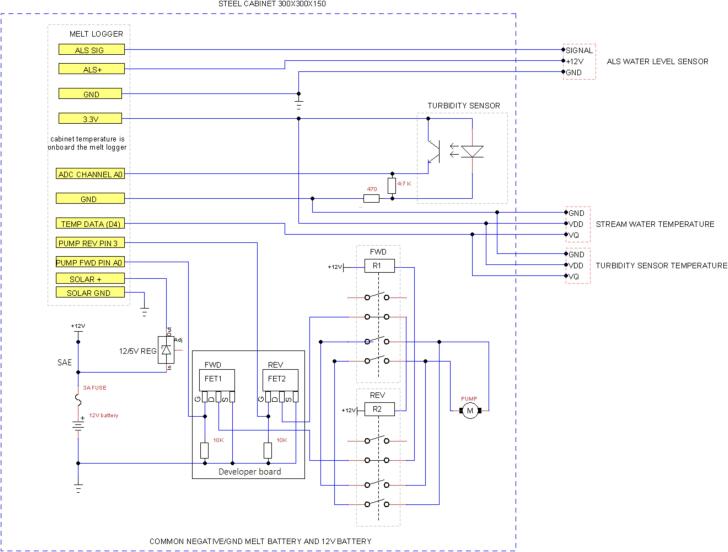
Electronic circuit schematic for the measurements. .

Source: https://github.com/Robwerg/MobileTurbidity

**Fig. 4:**
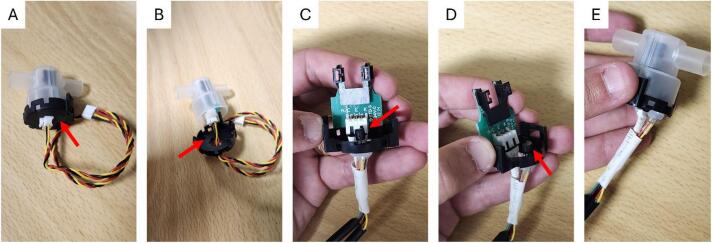
Adjusting the Turbidity Sensor (TST-10). A and B) Turbidity sensor before adding the temperature sensor. Red arrows indicate where the hole should be done. C and D) The red arrows point to the temperature sensor that has been added inside the turbidity sensor housing. E) Turbidity sensor ready to be installed into the cabinet. (For interpretation of the references to color in this figure legend, the reader is referred to the web version of this article.)

**Fig. 5:**
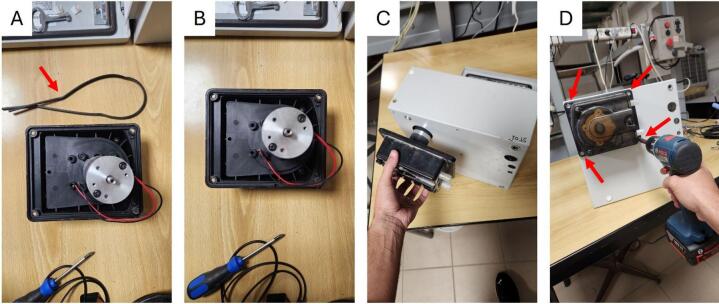
Installation of the peristaltic pump. The pump was installed outside of the back wall of the cabinet by passing the gear motor through the hole and fixing its case with 4 metal screws − 2.5 x 12 mm. A) The red arrow indicates the sealing gasket added to the pump to ensure that its assembly was sufficiently sealed. B) Shows the pump with the sealing gasket. C) Assembling the pump from the outside of the cabinet. D) The red arrows indicate where the pump is secured by the screws. (For interpretation of the references to color in this figure legend, the reader is referred to the web version of this article.)

**Fig. 6:**
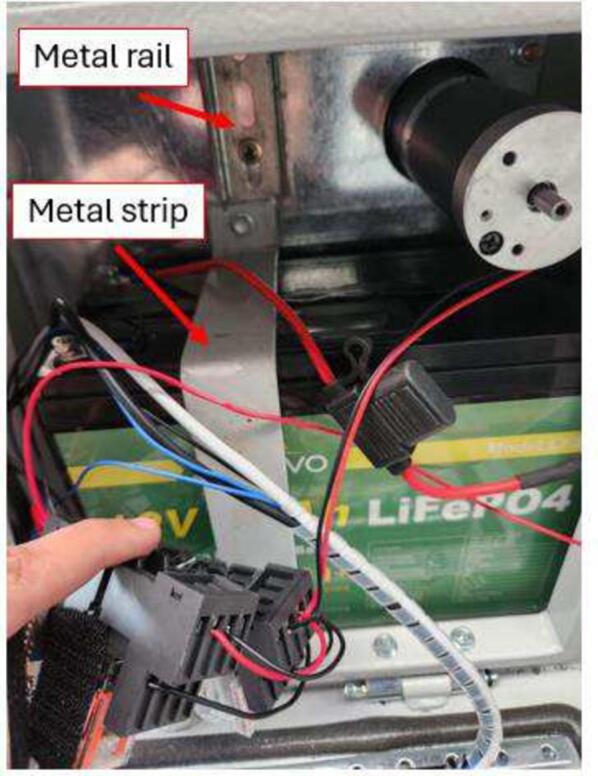
Installation of the 12 V lithium battery, and the metal strip and rail.

**Fig. 7:**
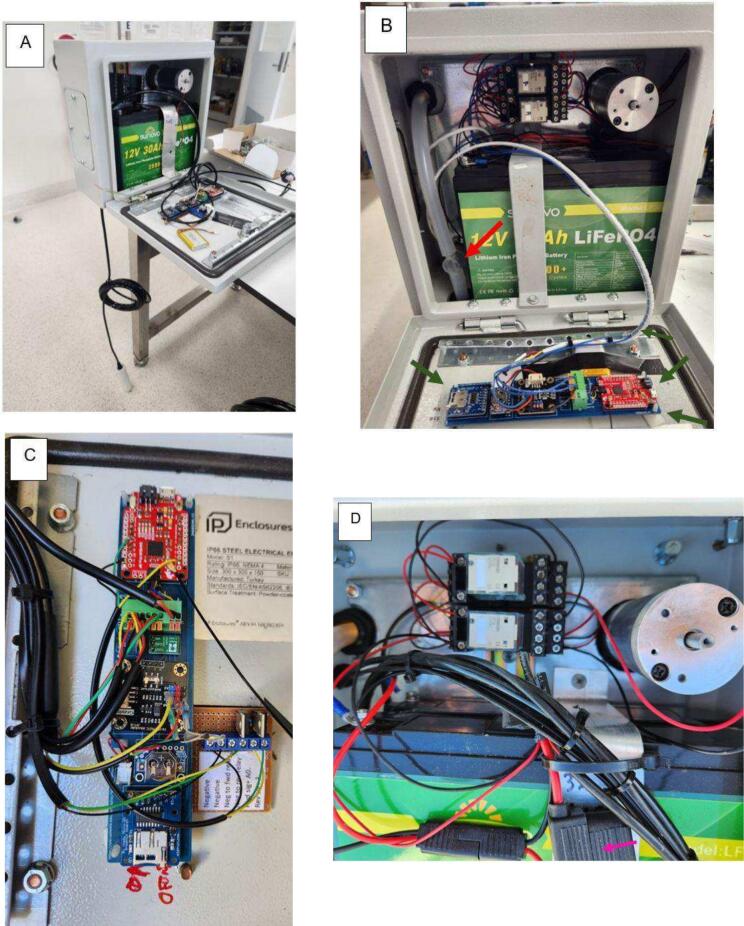
Installation steps. A) and B) Installing and fixing all the components of the monitoring station. The red and green arrows in “B” indicate where the turbidity sensor should be installed and how to fix the PCB with adhesive PCB mounts, respectively. C) and D) Zoom in on the connections of the electrical components as shown in Fig. 3. The pink arrow indicates where the 3A fuse should be inserted. (For interpretation of the references to color in this figure legend, the reader is referred to the web version of this article.)

**Fig. 8:**
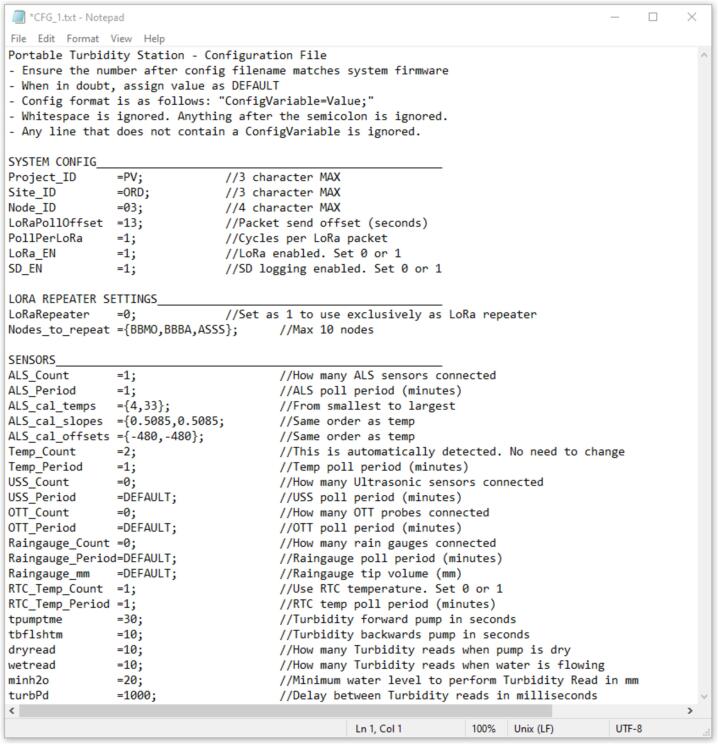
Configuration file. Each line includes comments explaining each variable directly on the same line, following the “//” symbol.

• *Electronic circuit schematic.JPG*: Electronic circuit schematic of the connections. Fig. 3 in this manuscript.
• *Portable_Turbidity_Station.ino*: this is the monitoring station Arduino script.
• *CFG_1.txt:* configuration text file to be loaded onto your SD Card. Fig. 8 in this manuscript.
• *IP66 enclosure – cabinet.JPG*: Initial stage of preparation of the monitoring station. Fig. 1, Fig. 2 in this manuscript.
• *Turbidity sensor housing:* Adapting the turbidity sensor by adding temperature sensor. Fig. 4 in this manuscript.
• *Assembling:* Final stage of assembly of the monitoring station. Fig. 6, Fig. 7 in this manuscript.
• *Pump assembling.JPG – Adding the pump to the cabinet.* Fig. 5 *in this manuscript.*
• *Assembling –* Supplementary Information*. pdf:* this is a supplementary file with a very detailed section on how to assemble the station together.
• *Recommendation to avoid bubbles –* Supplementary Information*. pdf:* this file brings together all the recommendation to avoid bubbles during the turbidity measurements.
• *Software user manual – Configuration file –* Supplementary Information*. pdf:* this is a friendly guide of how to configure the monitoring station.

## 5 Bill of materials summary

**Table:** 

Designator	Component	Number	Cost per unit −AUD	Total cost − AUD	Source of materials	Material type
Steel Cabinet 300x300x150	Galvanised Steel IP66 Enclosure Grey	1	114.26	114.26	tradezone.com.au	Metal
Cable Gland	Cable Gland Nylon IP68 Halogen Free Black 25 mm	2	1.20	2.40	rexel.com.au	Nylon
Cable Gland	Cable Gland Nylon IP68 Halogen Free Black 16 mm	1	0.75	0.75	rexel.com.au	Nylon
Cable Gland	Cable Gland Nylon IP68 Halogen Free Black 12 mm	1	0.59	0.59	rexel.com.au	Nylon
Pump (M)	Peristaltic pump (Model 353 K/ZLX)	1	180.81	180.81	alibaba.com	Other
MELT logger	Melt v1.1 logger board	1	194.88	194.88	https://github.com/Robwerg/Logger/tree/main	Other
ALS water level sensor	water level sensor 2 m range, 1–5 V output, temperature compensated, vented for atmospheric compensation	1	49.95	49.95	ahqidian.en.alibaba.com	Other
Turbidity sensor temperature	DS18B20 1-wire temperature sensor	1	8.45	8.45	core-electronics.com.au	Other
Stream water temperature	DS18B20 waterproof temperature sensor	1	23.20	23.20	core-electronics.com.au	Other
Turbidity sensor	Turbidity sensor (TST-10)	1	10.91	10.91	digikey.com	Other
tube	silicone tube 10 mm ID 16 mm OD	2 m	22.45 (with 5 m)	9.00	castorama.fr	Polymer
R1 and R2	Power Relay, 4PDT, 12 VDC, 5 A, MY, Socket, Non Latching	2	14.69	29.38	octopart.com	Other
FWD and REV	Relay Socket, DIN Rail, Screw, 14 Pins, 5 A	2	14.31	28.62	octopart.com	Other
FET1 and FET2	logic level NPN mosfet	2	1.09	2.18	digikey.com	Semiconductor
adhesive PCB mounts	3- adhesive PCB mounts	3	0.91	2.73	au.rs-online.com	Polymer
Developer board	developer pcb board	1	1.66	1.66	digikey.com	Other
10 k	Resistors (10 k Ω – 0.5w)	2	0.85 (pack with 8)	0.21	jaycar.com.au	Semiconductor
4.7 k	Resistors (4.7 k Ω – 0.5w)	1	0.85 (pack with 8)	0.11	jaycar.com.au	Semiconductor
470	Resistors (470 Ω – 0.5w)	1	0.85 (pack with 8)	0.11	jaycar.com.au	Semiconductor
12 V battery	LiFePO4 lithium battery 12 V 30Ah	1	180.46	180.46	fr.eco-worthy.com	Other
3A fuse	3 A Fuse	1	1.25	1.25	jaycar.com.au	Other
Metal screws	Metal screws 2.5 x 12 mm	8	5.24 (pack with 35)	1.20	bunnings.com.au	Metal
SAE	SAE to SAE 12 V DC Power	1	13.95	13.95	jaycar.com.au	Composite
Metal strip	Metal strip	2 cm x 30 cm	27.48 (90 cm x 30 cm)	0.61	bunnings.com.au	Metal
Metal rail	Metal rail	3.5 cm x 6 cm	7.11 (1 m)	0.43	digikey.com	Metal
12/5V REG	Voltage regulator	1	11.95	11.95	jaycar.com.au	Other
**Total**	**−**	**−**	**−**	**870.05**	**−**	**−**

## 6 Build instructions

### 6.1 IP66 enclosure − Cabinet

A total of 5 holes of varying diameters are required to prepare the cabinet for assembling all the components of the monitoring station. Fig. 1 shows the location and details of each hole.

An aluminium strip should be prepared and fixed to the cabinet to hold the battery securely inside. Additionally, an aluminium rail must be fixed to the back wall of the cabinet to hold both switches (power relays). The position and dimensions of both structures are shown in Fig. 2. Both structures are fixed using metal screws. The material and width of both structures do not influence the performance of the equipment. In this study, materials already available from other projects were reused.

### 6.2 Electronics

The monitoring station has a custom PCB, where break-out boards have been added to form the low-cost datalogger, known as the MELT datalogger. Some of the main components of our datalogger are: i) a real-time clock, so that measurements can be properly recorded at the time of measurement; ii) a micro-SD card break-out, so that data can be recorded for later download; iii) a 16-bit analog-to-digital converter, which provides sufficient resolution for this monitoring station; iv) a 3–12 V DC boost converter for full operation of the water level sensor and the peristaltic pump; and v) a Sparkfun Pro RF microprocessor with SAMD21 LoRa chip, which provides long-range wireless communication with low power consumption. More details and information about the MELT datalogger can be found in James et al. [22].

The connections between the MELT datalogger and the other components of the monitoring station (peristaltic pump, forward and reverse power relays, temperature sensors, water level sensor, 12 V battery and turbidity sensor) can be seen in Fig. 3, which illustrates the electronic circuit schematic for the measurements.

### 6.3 Turbidity sensor housing

A 1-wire temperature sensor (DS18B20) is added into the turbidity sensor (TST-10) housing to enable temperature compensation of turbidity readings (mV) with the sensor housing temperature at the moment of the measurements (Fig. 4). The sensor is installed using a hole (with a size of the temperature sensor diameter) on the top plastic of the turbidity sensor (black part), and then passing the wire temperature sensor through it. After this process, glue is added to the outside to better fix the wires and make the connection more robust.

### 6.4 Assembling

The first step is to add the four cable glands to the cabinet, two cable glands of 25 mm for the sampling tubes, one of 12 mm for the cable of the temperature sensor, and one of 16 mm for the cable of the water pressure sensor. The positions of the holes are shown in Fig. 1. The cable glands used in this station are appropriately sized to fit the diameter of the intake tube as well as the temperature and water level sensor cables. As a result, no additional sealing elements were required during the assembly of these components.

The next step is to fix the peristaltic pump (model 353 K/ZLX) to the external part of the cabinet with four metal screws (Fig. 5). A sealing gasket was added to the pump to ensure that its assembly was sufficiently sealed, preventing water leakage into the monitoring station. The gear motor should be put inside the cabinet by passing it through the hole (from the outside to the inside), so the electrical part of the pump is protected from the weather. This design is desirable because it prevents any possible leakage from the pump from reaching the electronics of the monitoring station.

The 12 V lithium battery with a 30Ah capacity can be placed on the bottom wall within the cabinet. We chose to use lithium batteries because they are relatively lighter and offer high energy density, extended lifespan, and fast recharging, making them ideal for our monitoring station. The next step is to fix the aluminium strip and rail inside the cabinet (Fig. 2). Importantly, the battery must be held by the metal strip (Fig. 6) to prevent it from moving and damaging other components or their connections.

It is recommended that the MELT datalogger be located inside the box lid, which will facilitate its operation and maintenance during *in-situ* monitoring. The datalogger can be fixed using adhesive PCB mounts (Fig. 7B). High temperatures may compromise the adhesive's effectiveness, especially in a metal enclosure, which can retain and conduct heat more efficiently. If the temperature inside the station becomes too high, the adhesive may lose its bonding strength, potentially causing the datalogger to detach. To mitigate this risk, mechanical fasteners or heat-resistant adhesives specifically designed for high-temperature environments could be used, ensuring the stability of the datalogger under such conditions.

The sampling tube (silicone tube) can be installed by passing it through the cable glands. The turbidity sensor should be installed in the middle of the sampling tube, within the cabinet (Fig. 7B).

Both switchers (power relays) should be placed on the metal rail (Fig. 7D). The 3A fuse must be installed to protect the entire system from possible overload, as shown on the electronic circuit schematic (Fig. 3).

The cables of the water pressure sensor and temperature sensor should be passed through their designed cable glands (from the outside to the inside) to connect them to the MELT datalogger, leaving both sensors outside of the cabinet.

To finish assembling the station, connect all other components as shown in Fig. 3. Fig. 7 shows the mobile turbidity monitoring station after all its components have been assembled. For further details on the station assembly, please refer to the Supplementary Material.

As this turbidity sensor is optical, it must be positioned inside the cabinet, thus avoiding interference from ambient light. The sensor should be installed in a vertical position to avoid air accumulation, which could affect turbidity measurements. Furthermore, the sampled water passes through the turbidity sensor before passing through the peristaltic pump, which avoids pressure changes and increased turbulence when measuring turbidity. Turbidity readings are only taken once the intake tube is filled with sample water and free of air bubbles, ensuring greater reliability in the measurements. Additional recommendations on how to prevent air bubbles from affecting turbidity readings can be found in the Supplementary Material.

It is recommended that all cables be secured with cable ties, to avoid movement and possible breakage during the opening and closing of the cabinet. Additionally, special attention must be paid to the extensions of the wires that connect the station components, as the distance between the components can be changed depending on whether the cabinet has its lid open or closed. When closing the cabinet, pay attention to whether the metal strip, which holds the battery, is touching any board or electrical connection, as this could cause a short-circuit, resulting in damage and potential fire. A non-conductive coating, such as Plasti Dip®, can be applied to cover part of the metal strip, thereby preventing any short circuit issues in case of contact with the electronics.

## 7 Operation instructions

***Step 1: load the code and configure the station***

Hardware operation begins in the laboratory. The Arduino code [23] needs to be loaded onto the SparkFun SAMD21 Pro RF microprocessor board. Thus, connect this board to a computer and, with the Arduino IDE software, load the code onto the board.

A micro-SD card must be inserted into the micro-SD break-out board. Inside the micro-SD card, there is a configuration file (CFG_1.txt, Fig. 8) that brings together all the needed settings to prepare the station as desired. The file must be filled out with the parameters for water level calibration and: i) the adding information on how long the pump will run in forward and reverse modes, ii) the triggering of the water level to activate the pump, iii) the period between each measurement cycle, iv) the name of the station, v) the number of each sensor, vi) the number of measurements in dry and wet conditions, and vii) the delay between each measurement.

The configuration file “CFG_1.txt” can be easily edited using any text editor, such as Notepad. This file includes comments explaining each variable directly on the same line, following the “//” symbol. These comments are ignored by the program but serve to facilitate understanding of each variable’s purpose. Further details on how to configure this configuration file can be found in the Supplementary Material.

**Step 2: *sensors calibration***

Calibration of the water level sensor should be done under laboratory conditions. Calibration can be done using a linear equation; using at least 2 points with varying water levels should therefore be sufficient for this. It is mandatory that you calibrate the sensor in the same orientation as it will be deployed in the field.

The turbidity sensor calibration needs to be done in two parts. The first consists of fitting a linear model between the sensor housing temperature and the turbidity measurements (in millivolts, mV) for temperature compensations, and the second part consists of calibrating the sensor for the parameter of interest, for example, turbidity (NTU/FNU) or SSC (g/L).

The first part is done in the laboratory, using samples with known concentration or turbidity. The methodology used for preparing these known concentration samples is further detailed in Section 7. The effect of temperature on turbidity measurements (mV) can be verified by increasing or decreasing the sample temperature throughout the experiment, across the range expected for the monitoring location. A practical method that can be used is to refrigerate samples then measure their turbidity while allowing them to come to room temperature. Or even, using equipment that allows you to heat the sample more quickly, e.g. magnetic stirrer with hot plate. It is recommended that the temperature, and the turbidity or concentration ranges of the samples used in this calibration is representative of that expected for the monitoring location. The average between each slope of the linear models should be used for temperature compensation.

The second part can also be done in the laboratory before installing the field monitoring station, but this is not mandatory. If uncalibrated, raw voltage measurements can be used and related to the parameter of interest as measured from field samples. These calibration processes will be further discussed in the next section.

***Step 3: Determination of pumping time***

To ensure accurate turbidity readings of the water samples pumped by the peristaltic pump, it is crucial to determine the appropriate duration for which the pump needs to operate. The required pumping time is directly dependent on the diameter and length of the intake tube.

Therefore, following the installation of the monitoring station, it is recommended to conduct a preliminary test to accurately determine the time required for the water sample to fully fill the sampling system without the presence of air bubbles. This test will ensure that the system is completely primed and free of bubbles prior to conducting turbidity measurements, thereby ensuring the accuracy of the obtained readings.

***Step 4: Station installation in the field***

**Fig. 9:**
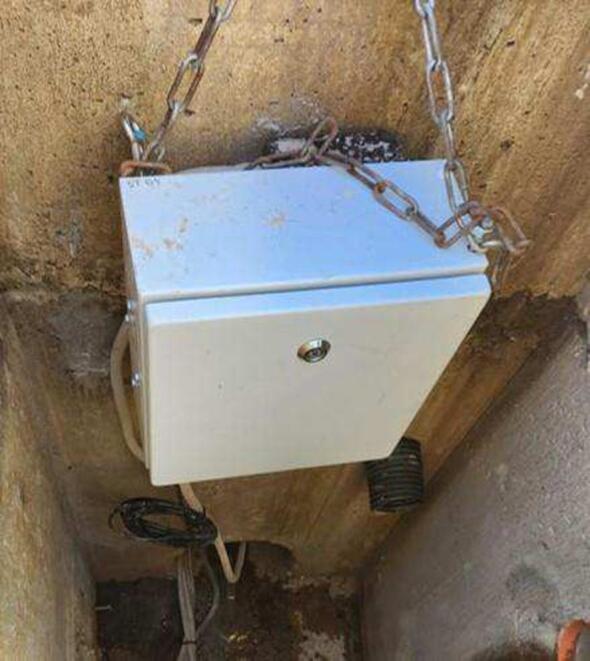
Monitored station suspended by chains on a wall inside a stormwater drain.

After carrying out these procedures in the laboratory, the monitoring station is ready to be deployed. The first step is to install the cabinet in a safe location (for example, away from flooded areas or any area where the station could be submerged in water, as the cabinet is not waterproof). The ideal is to attach it to a wall or post (Fig. 9). The cabinet is water resistant, so there is no problem installing it in an open area, but it is recommended to install the monitoring station in a covered location, thus protecting it from rain and direct sunlight. Sunlight can heat the cabinet and therefore affect the turbidity sensor housing. Our experiments have shown that situations where the difference between the sensor housing and water temperatures is approximately 7 °C or more, condensation can form on the inner wall of the sensor housing, which causes the sensor to not function properly due to optical disturbance. Although not tested in this study, the addition of a desiccant to the turbidity sensor enclosure may be a potential solution to prevent or minimize the formation of condensation on the inner wall of the sensor housing.

The orientation of the water level sensor plays a key role in the data collection. For example, if it is oriented against the water flow, the speed of the water could cause overpressure in the sensor, which would consequently cause measurement errors. Therefore, the orientation of the water level sensor depends on the monitored flow conditions. For most cases, it is recommended to place the sensor in a vertical position (consequently, perpendicular to flow direction), thus avoiding overpressure caused by the flow speed. The vertical position corresponds also to a horizontal position of the sensor membrane, thus reducing measurement error. Furthermore, the sensor must be securely fixed, as any change in orientation or positioning may lead to an incorrect reading.

The sampling tube must be placed in a location where the data collected represents the entire flow that passes into the monitoring area. Typically, the middle of the cross section is desired. The depth from the lowest point in the cross-section depends on the environment being monitored; in environments with intermittent flow the inlet may be placed close to the invert to maximise data collected, while in continuously wetted environments the sampling position should be selected based on the expected vertical concentration gradient to ensure the representativeness of the suspended sediment concentration of the cross section [23], [24]. The orientation of the tube must be approximately vertical, from the cabinet to the monitored point, to avoid both sedimentation and air accumulation inside the tube. The latter is particularly important, as microbubbles can mimic sediment, causing the turbidity sensor to measure incorrectly.

The monitoring station outlet tube must be placed either in a bottle, to collect samples during the monitored flow, or facing the flow direction, downstream of the location where samples are collected. This way, the water sample can return to the flow without disturbing the sampling area. It is extremely important that the outlet tube is not submerged in water due to the operation of the station. The tube outlet needs to be in the air so that when the pump is turned on in reverse mode, the entire system can be purged, and the sensor left in the air, so that it does not remain permanently wet. This can extend the effective operating life of the sensor by preventing sediment from sticking to the sensor and algae growth inside it.

Although no biofouling was observed in the intake tubing during this study, if its formation is detected or considered likely, it is recommended to replace the intake tubing or use a dark PVC conduit to encase the intake tubing, protecting it from sunlight exposure. In any case, it is likely that any algae or biofilm dislodged from the tubing during operation would be flushed out at the start of the pumping cycle, as the pump operates for several seconds before turbidity measurements are taken. This flushing process minimizes the risk of contamination from biofouling, ensuring the integrity of the turbidity measurements.

***Step 5: Collecting the measured data***

The entire data set is recorded on a micro-SD card, reducing the need for frequent visits. However, we recommend regular visits to the site to download the measured data and check that everything is working as planned. First visits could for example be used to fine tune the monitoring settings. Before collecting the micro-SD card and downloading the data, be sure to turn off the station, otherwise the micro-SD card may become corrupted, causing the recorded data to be lost. Then re-insert the micro-SD card and turn on the station so it is ready for the next measurements.

Removing the memory card to collect data does not affect the station's settings, as the configuration is stored in the CFG_1.txt file. Therefore, when the station is restarted, it automatically resumes operation with the same settings specified in this configuration file. This ensures that the system continues to operate consistently according to the originally programmed parameters.

As the monitoring station uses a LoRa system, a gateway can be used to receive newly measured data when near to the station. This helps to check the measured values of water level, turbidity and temperatures, and also whether the station is working, without having to disturb the micro-SD card.

Quantifying the distance required for data transmission to the gateway was beyond the scope of this study. However, this distance is variable for each specific experiment setup, as it depends on several factors, such as the location where the station is installed (whether in an open or confined area) and the presence or absence of natural or artificial structures in the surroundings (e.g., buildings, trees, or houses).

It is important to note that the LoRa configuration provided here in the code uses the appropriate frequency for Australia. Therefore, an assessment must be made of the frequency permitted by the jurisprudence of the country/location where the monitoring station is going to be used.

Battery voltage should be measured at each visit to the monitoring site to avoid battery depletion, which would otherwise result in the station's inability to operate the pump and obtain adequate turbidity measurements.

It is also recommended that every time the station is visited, the water level is measured manually. Sensor drift (change in reading for a stationary phenomenon) is common in many sensors, and has been confirmed to occur in the water level sensor used for this turbidity monitoring station, in situations where water flow is intermittent. Therefore, it is important to regularly check and update the water level sensor calibration parameters (offset) in the configuration file, to better reproduce observed water level conditions. Information about offset changes can also be of great value in post-data analysis.

## 8 Validation and characterization

### 8.1 Laboratory experiments

Three tests were carried out under laboratory conditions before installing the station in the field: i) to calibrate the water level pressure sensor; ii) to verify and measure the importance of temperature compensation in the accuracy of the turbidity sensor; and iii) to verify the performance of the monitoring station in monitoring the concentration of fine suspended sediments.

**Fig. 10:**
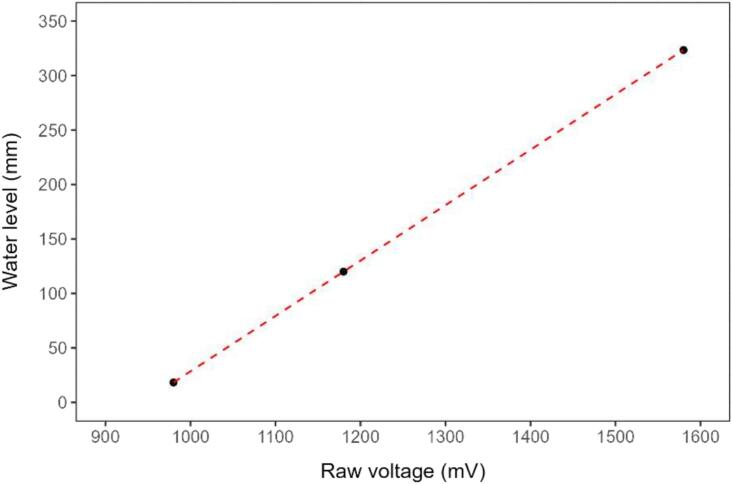
Water level sensor calibration.

For the water level calibration, a transparent plastic tube of 20 cm in diameter was used in a vertical position with a ruler to calibrate the sensor. Three different water levels in the tube (Fig. 10), covering the expected measurement range were used to calibrate a linear regression equation (eq.1):

(1) $$Waterlevel\left(mm\right)=0.5085*Rawvoltage\left(mV\right)-480$$

Under operation procedures (Section 6), linear calibration can be achieved with two points, but three points were used here to confirm linearity during the development of the system. Water temperature was not considered in the calibration of the water level sensor, as the temperature variation, according to the manufacturer, represents +-0.01 % FS/°C, which is within sensor accuracy (0.2 % FS − 0.5 % FS). During the laboratory experiments, six water pressure sensors were calibrated, and their slopes and intercepts varied from 0.5058 to 0.5110 and from −480 to −492, respectively. Therefore, each sensor should have their own calibration.

The calibration covering up to a depth of 300 mm was adequate for the scope of this study because the drainage pipe where the system was installed has a diameter of 300 mm. Furthermore, each sensor requires individual calibration, meaning that the specific calibration values obtained in this study will not be directly applicable to other installations or users. Nonetheless, the calibration methodology employed can be easily replicated, and the expected maximum water levels for the deployment should be used in the sensor calibration. However, it is important to note that the sensor's maximum measurement range is 5 m (manufacturer specification), which should not be exceeded. This ensures that the system remains flexible and applicable to various field conditions.

Calibration of the turbidity sensor was performed using samples with known concentration, so that the turbidity measurements (mV) were directly related to the sample concentration (g/L) and not to Formazin Nephelometric Units (FNU) nor to Nephelometric Turbidity Units (NTU).

**Fig. 11:**
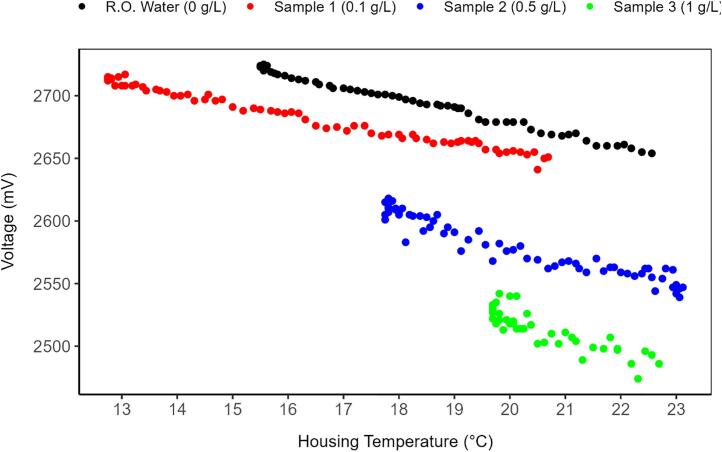
Turbidity sensor measurements for different concentrations and a range of temperatures.

Initial testing showed that turbidity sensor voltage readings for a given sample varied linearly with temperature. To test the required temperature compensation for turbidity measurements, four laboratory samples (mix of bentonite crushed and sieved to <63 μm and tap water) with known concentrations of 0, 0.1, 0.5, and 1.0 g/L were manually prepared and used in the laboratory experiment. For each concentration, the sensor housing temperature was varied gradually from 13 to 23 °C over a total of approximately 50–60 measurements (Fig. 11). All data collected during the experiments (n = 231) were randomly divided into training (90 %) and testing (10 %) datasets. Turbidity values (mV) were adjusted for temperature using the following equation (eq.2).

(2) $${\mathrm{Turb}}_{\mathrm{Adjusted}}=Turbidity-\left[{\mathrm{Slope}}_{\mathrm{average}}*\left({\mathrm{Temp}}_{\mathrm{Housing}}-{\mathrm{Temp}}_{\mathrm{Reference}}\right)\right]$$

Where: ${\mathrm{Turb}}_{\mathrm{Adjusted}}$ is the turbidity adjusted by the housing temperature (mV), Turbidity is the turbidity (mV) measured by the turbidity sensor, ${\mathrm{Slope}}_{\mathrm{average}}$ is the average slope of the four linear regression lines for each concentration (−7.96; −9.60; −11.35; and −14.62 mV/°C) between the turbidity measurements (mV) and the housing temperature. The average slope was −10.88 mV/°C, ${\mathrm{Temp}}_{\mathrm{Housing}}$ is the temperature measured inside the turbidity sensor housing, ${\mathrm{Temp}}_{\mathrm{Reference}}$ is the reference temperature. For this case, a temperature of 20 °C was chosen as this temperature is common in all concentrations tested here.

**Fig. 12:**
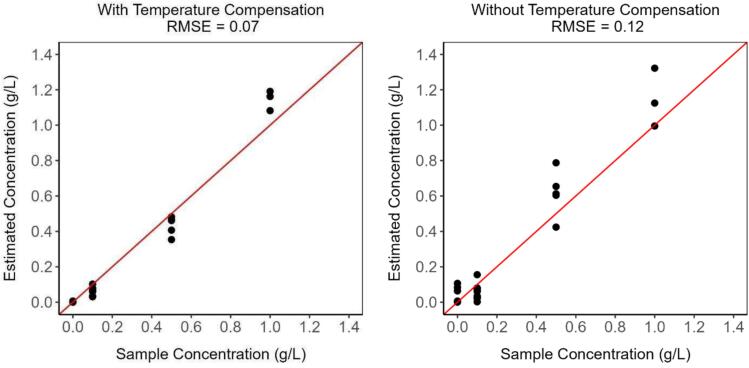
Laboratory results. On the left, the results obtained for the estimated concentration after applying the temperature compensation. On the right, the same results, but without applying the temperature compensation. The red line indicates the 1:1 line between estimated concentration and sample concentration. N = 23. (For interpretation of the references to color in this figure legend, the reader is referred to the web version of this article.)

Temperature compensation was found to greatly improve measurement accuracy. Fig. 12 presents two graphs with the estimated concentrations and the concentration of laboratory samples for both scenarios with and without temperature compensation. The root-mean-squared error (RMSE) for the testing dataset reduced from 0.12 g/L, without temperature compensation, to 0.07 g/L, with temperature compensation.

### 8.2 Field validation

**Fig. 13:**
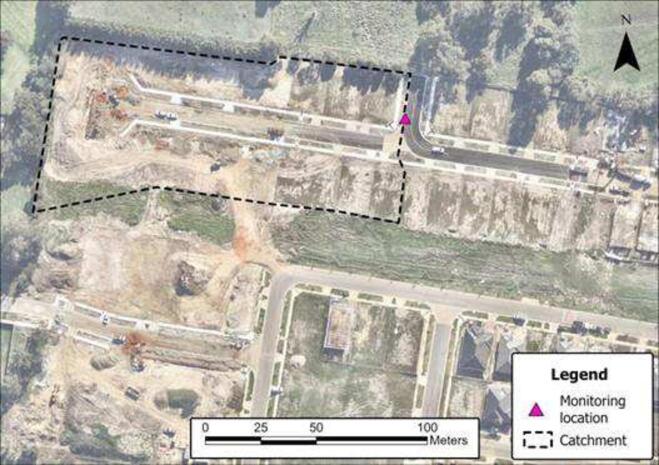
Monitoring catchment in Melbourne, Australia. Image obtained free of charge from Nearmap US Inc.

For field validation, a small catchment (1.1 ha – Fig. 13) undergoing urbanization in Melbourne, Australia, was chosen. The monitoring station was installed in the underground drainage system, inside a stormwater pit. It monitored the water level and suspended sediment concentrations of the runoff from this small catchment for six months (18/05/2023 – 17/11/2023).

**Fig. 14:**
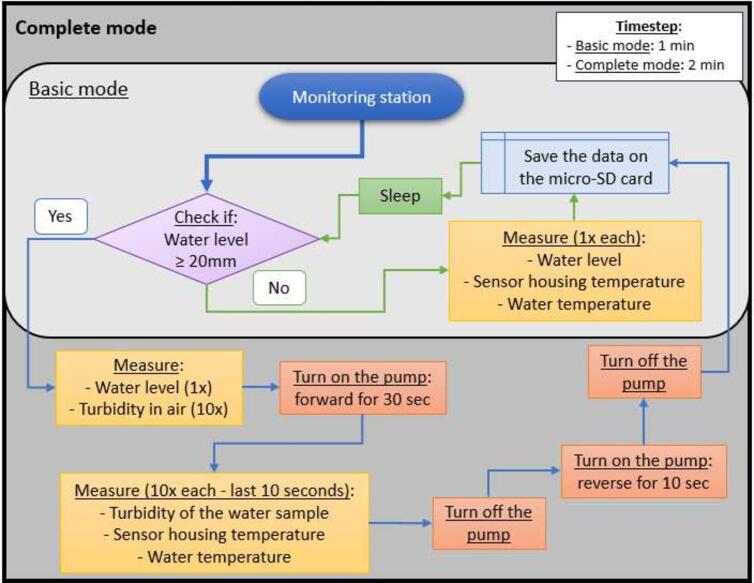
Flowchart of the two modes (basic and complete) of the monitoring station operation. The duration of the sleep function is always calculated using information from the updated RTC and the period between each measurement cycle defined in the configuration file.

The monitoring station was configured using two measurement modes: basic and complete. The basic mode has a 1-minute timestep and consists of measuring and recording on the micro-SD card the water level, water temperature and sensor housing temperature (but not turbidity) every minute. Once a specified water level threshold is reached (20 mm in the example in Fig. 14), the program calls the complete mode. This mode encompasses the basic mode and additionally measures the turbidity of the air (dry conditions) and the water sample (wet conditions).

The duration of the sleep function is dynamic. In other words, it is always calculated using information from the updated real-time clock (RTC) and the interval (period between each measurement cycle) defined in the configuration file.

The complete mode has a 2-minute timestep, and consists of first measuring and recording the water level (once) and the air turbidity (ten times), and after that, a peristaltic pump draws water from the monitoring flow through a silicone tube, upwards into the sampling enclosure, through the turbidity sensor, exits the sampling enclosure, through the peristaltic pump, past a waterproof temperature sensor and then returns to the monitoring flow.

In complete mode, the station was configured to operate the pump for a total of 30 s per sampling cycle. The first 20 s are dedicated to priming the system, ensuring that the intake tubing is fully filled with water and free of air bubbles before any measurements are taken. This duration was determined to be sufficient based on the characteristics of the sampling system and the expected flow rate of the peristaltic pump under field conditions.

After this initial 20-second priming period, the station proceeds to take 10 measurements of water turbidity, water temperature, and sensor housing temperature, recorded at one-second intervals during the final 10 s of the pumping cycle. This approach ensures that the measurements reflect a fully representative water sample. Following the measurement phase, the pump is reversed for 10 s to purge the system, removing any residual water from the sampling system, thereby preventing potential biofouling and ensuring the sensor remains in air for subsequent cycles. The following flowchart shows the operation of the monitoring station (Fig. 14).

A total of 207,142 water level and 5,893 turbidity measurements were recorded with the monitoring station. A total of 16 water samples were also manually collected during the monitored period and their total suspended solids (TSS) concentration analysed in a NATA-accredited (National Association of Testing Authorities) laboratory.

**Fig. 15:**
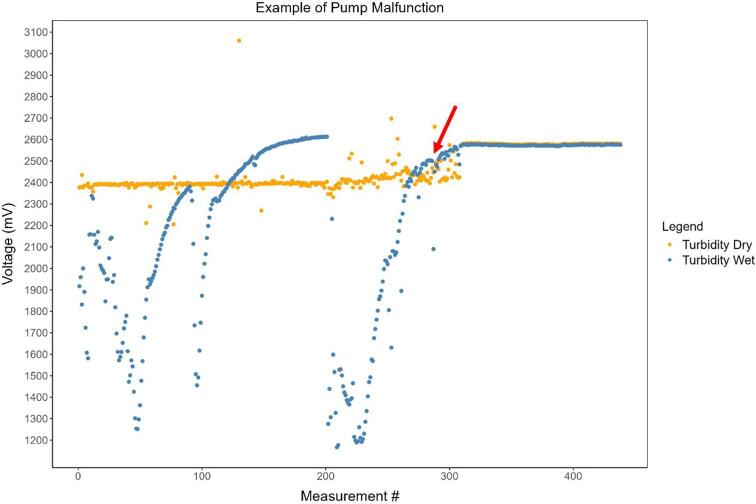
Example of pump malfunction. Air turbidity (dry conditions) is shown in orange and water turbidity in blue. It can be easily seen that there is a problem with the monitoring station from around measurement number 300 (red arrow), which was caused by battery exhaustion. The pump started to work and halfway through the pump cycle the battery became discharged and the pump stopped working. Consequently, we can observe that the values referring to voltage measurements for dry and wet conditions are identical. (For interpretation of the references to color in this figure legend, the reader is referred to the web version of this article.)

Turbidity measurement in dry conditions is a mechanism to identify station issues and also help with data post-processing. For example, if the sensor starts to become clogged by sediment deposits or biofouling (e.g. algal growth), the turbidity in the air measurements will change over time and this will show when these problems started to happen, allowing decisions to be made in selecting data periods for inclusion or exclusion. Furthermore, quantifying these changes in turbidity in the air measurements over time can allow correction of turbidity measurements from water samples. Another use of turbidity measured in air is to identify periods in which the pump is not working correctly, as turbidity in dry conditions will have the same values as the turbidity when the pump is not functioning (Fig. 15).

Turbidity measurements are highly dependent on sediment type, density, size, shape, color [10], [25]. A variation in these characteristics can lead to changes in the relationship between total suspended solids and turbidity measurements [26]. These observations have been verified and validated here. The model developed and calibrated with laboratory samples (mix of bentonite and tap water) was applied to estimate the suspended sediment concentration of all these 16 field samples after adjusting the raw turbidity measurements using the temperature compensation model (Equation (2). A RMSE of 24.71 (g/L) was found, indicating an unacceptably high measurement error.

**Fig. 16:**
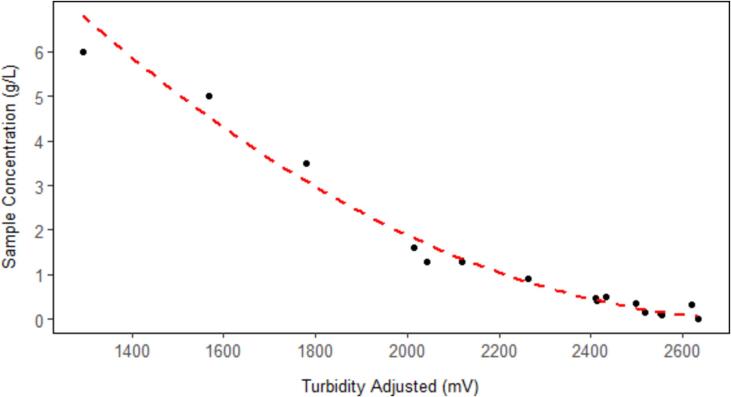
Plot between the adjusted turbidity, after applying the temperature compensation model (Equation (2), and the TSS values of the water samples collected in the field during the monitoring period. R^2^ = 0.97.

This shows that the calibration process must be carried out with site-specific samples. Thus, the TSS and related turbidity measurement of the 16 field samples were used to calibrate the turbidity sensor under field conditions (similar to development of a turbidity-TSS rating curve). The raw turbidity measurements were first adjusted by the temperature compensation model (Equation (2), and then plotted (Fig. 16) against the TSS of all samples to establish a second-order polynomial regression model (Equation (3), R^2^ = 0.97).

(3) $$Conc\left(g/L\right)=3.05e^{-6}*{{\mathrm{Turb}}_{\mathrm{Adj}}}^{2}-0.017*{\mathrm{Turb}}_{\mathrm{Adj}}+23.62$$

To validate the model, leave-one-out cross-validation (LOOCV) method was used. The entire dataset (n = 16) was repeatedly split into training and testing sets, and each sample was used alone once as a testing set while the remaining samples formed the training set. The final RMSE value was calculated by taking the average of the 16 RMSE values (1 for each of the 16 models).

**Fig. 17:**
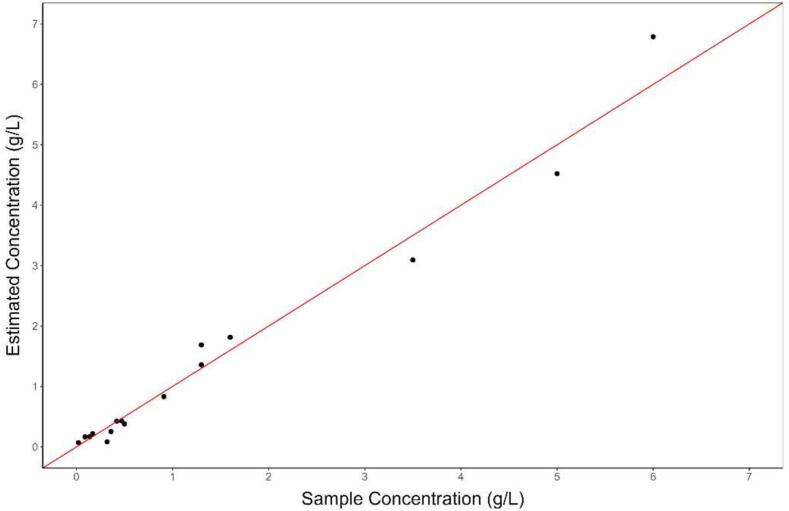
Results using the field samples. The red line indicates the 1:1 line between estimated concentration and sample concentration. n = 16. RMSE = 0.32. (For interpretation of the references to color in this figure legend, the reader is referred to the web version of this article.)

The estimated concentration for all samples using the model developed with field data produced a RMSE of 0.32 g/L (Fig. 17), which is two orders of magnitude lower when compared to the RMSE produced by the laboratory model (24.71 g/L), when using samples collected *in-situ*. This level of accuracy was considered adequate for the monitoring location, where sample concentrations ranged from 0 to 6 g/L. Therefore, it is highly recommended that the calibration process be carried out using samples from the target monitoring area, as also proposed by a number of other authors [18], [27], [28].

The calibration process can either be performed as it was in this study, by collecting multiple samples during the monitoring period, analysing them for total suspended solids concentration, and using these values to calibrate the turbidity sensor. It can also be done by collecting a large sample from the monitoring site to prepare some small sub-samples with different concentrations, and, from there, carry out the sensor calibration process in the laboratory.

**Fig. 18:**
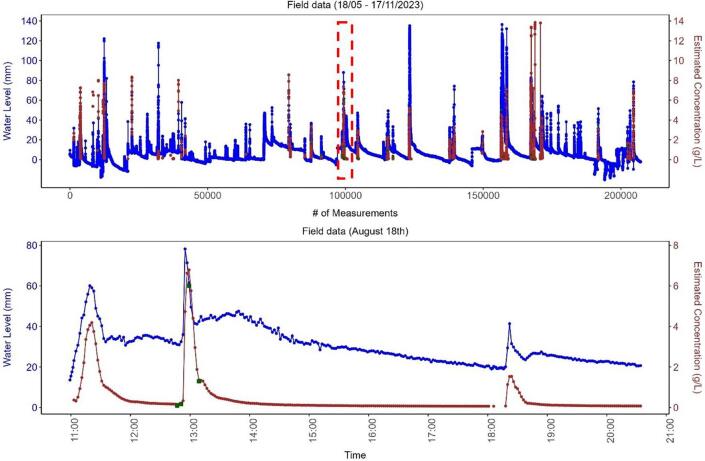
Results of the TSS estimated by the regression model built between the turbidity measurements (mV) and TSS concentration from the field samples. A) Suspended sediment concentration (g/L) estimated for the entire monitoring period. The water level is shown in blue on the primary Y-axis, and SSC on the second Y-axis. The SSC (g/L) estimated is shown in red, and the green squares represent the TSS concentration of the samples collected during fieldwork. The red dotted square in red represents the period shown in Fig. 18B. B) An event where 4 samples were collected. The water level is shown in blue on the primary Y-axis, and SSC on the second Y-axis. The SSC (g/L) estimated is shown in red, and the green squares represent the TSS concentration of the samples collected during fieldwork. (For interpretation of the references to color in this figure legend, the reader is referred to the web version of this article.)

After applying the regression model developed with the field data, it was possible to estimate the suspended sediment concentrations for the entire monitored period, which is shown in Fig. 18A. A rain event that occurred on August 18th is shown in Fig. 18B. It shows that the sensor had a strong signal response from catchment runoff and with a few samples (n = 16, and TSS ranging from 0.1 to 6 g/L) it is possible to calibrate the turbidity sensor to estimate the TSS for the entire data series.

As the study area is very small with high impervious cover (∼1 ha), it is expected that the hydrological response of the catchment would be very rapid. In other words, when the precipitation event ends, the water level is expected to return to zero relatively quickly, as there are no other significant water inputs in the basin.

However, as shown in the figure, we observe that the water level takes longer to return to zero, and in some cases, there is even a slight increase after the peak. This can be explained by observations made during fieldwork, where runoff from a neighbouring agricultural property entered the same drainage system where our monitoring equipment was installed. As a result, we see a slower decrease in water levels, and in some instances, a small secondary peak between the three monitored peaks. Effectively, there are two runoff processes occurring in this catchment – quick runoff from roads and neighbouring construction areas, and slow runoff from agricultural areas.

The fact that the sediment concentration does not follow the water level in the secondary peaks can be attributed to the fact that rural areas typically have less exposed soil compared to construction areas, which are more prone to erosion. In construction sites, surface runoff during intense rainfall events tends to carry a higher concentration of suspended sediments compared to runoff from rural areas (1 or 2 orders higher – Russell et al., 2017) [29].

### 8.3 Monitoring station characterization

The monitoring station presented here works with four sensors: one turbidity sensor, two temperature sensors (sensor housing and water), and one water level pressure sensor. The station works in two different modes, basic and complete (Fig. 14), depending on the water level of the flow and trigger level set for the monitoring program. The timestep for the basic and complete modes can be defined in the configuration file to suit different monitoring needs. The basic mode measures the water level, and the temperatures of the water and the sensor housing, but not turbidity; it has a lower energy consumption compared to the complete mode because the pump is not operating. The complete mode encompasses the basic mode and additionally measures turbidity, in dry and wet conditions. The implementation of the two modes allows for monitoring of intermittently flowing water without unnecessary battery usage and wear and tear from continual pump operation.

A total of 10 turbidity measurements for both wet and dry conditions are taken in each pump cycle by default, but the number can be changed as desired in the configuration file. Using the median value of the measurements helps to eliminate unwanted data spikes caused by bubbles (at the risk of missing some of the larger sediment particles).

In dry conditions, the turbidity measurement works as an indicator of whether the turbidity sensor requires maintenance, as a reduction in measured voltage would indicate that the sensor requires cleaning, possibly due to fouling. Additionally, quantifying these changes in turbidity in air measurements over time can allow correction of turbidity measurements from water samples. Furthermore, it also serves to indicate whether the pump is working correctly, as the measured voltage in wet and dry conditions would be the same if the pump were not operating.

The turbidity sensor is inside an IP66-rated cabinet for environmental protection, so the station also requires a peristaltic pump to run the water through the turbidity sensor. As it is an optical sensor, the darkness provided by the cabinet is essential to avoid the interference of the ambient light of the measurements, and it is also a mechanism to avoid algae growing on the sensor. These are the main problems reported for turbidity measurements in water bodies [12], [13], [28].

The peristaltic pump used at our monitoring station is similar to the one used by the Hach Sigma 900 Max automatic sampler, which makes it possible to compare the samples collected by both systems. Furthermore, our station allows water samples to be collected similarly to the samples collected by the automatic sampler for subsequent analysis in the laboratory, e.g. gravimetric analysis for TSS.

However, this monitoring station can be adapted with smaller pumps if considered appropriate for the monitored environment. This would also allow the selection of smaller battery and cabinet, which would significantly reduce the cost of the monitoring station, considering that these three elements (pump, battery and cabinet) represent 55 % of the total cost of this monitoring station.

The entire turbidity sampling system is positioned in the vertical direction, to avoid the accumulation of bubbles within the system (tubes and sensor), which can influence the turbidity readings. Additionally, the turbidity sensor is positioned before the pump, which also minimizes the effect of bubbles on turbidity readings as reported by Gillett and Marchiori (2019) [12].

The station has two activation modes for the pump: forward and reverse. The forward mode is activated when the trigger level is reached, and runs the water through the turbidity sensor for the turbidity measurements. The reverse mode works as a safety mechanism for the turbidity sensor, as it purges water from inside the turbidity sensor and the sampling tube, leaving them in the air, which prevents the entire internal sampling system from being in contact with water for long periods of time. This reduces the likelihood of sediment sticking to the inner wall of the turbidity sensor and additionally prevents algal growth. Therefore, the reverse mode increases the life cycle of the sensor, which reduces the number of times it needs to be cleaned. For the present experiment, it was not necessary to clean the turbidity sensor at any time during the 6 months of monitoring. The protection from ambient light to avoid algae and the reverse mode significantly reduce maintenance (cleaning) requirements.

The station is set to sleep mode between measurements, increasing autonomy and battery lifespan. The frequency of battery changes depends on how the station is configured to perform turbidity measurements. In other words, the pumping time (forward and reverse modes) as well as the frequency of turbidity measurements directly influence the station's autonomy.

For this study, it was noted that the battery was sufficient to drive the pump for around 700 pumping cycles, which represents, for this study case, continuous monitoring of turbidity every two minutes and water level and temperature every minute for approximately five weeks, assuming the pump is activated for five hours per week.

An important limitation of the monitoring station is the measurement of turbidity in situations where the temperature of the turbidity sensor housing differs from the water temperature by about 7 °C or more. Situations like this can cause condensation on the inner wall of the turbidity sensor housing, which causes the turbidity sensor to read incorrect values. This limitation should be better explored in future studies, by finding a way to better insulate the sensor housing against condensation or adding protection to the cabinet that can control the device's internal temperature. Additionally, although not tested in this study, incorporating a desiccant within the turbidity sensor housing could potentially minimize or even prevent condensation formation.

There are some other improvements that could be made to the station. These improvements include changes to the program code and the addition of new components to the monitoring station. For the code, it may be possible to use machine learning anomaly detection methods to identify and notify the user of periods when the station is not working properly (e.g., battery exhausted, pump not working). Other possible improvements are based on adding new components to the station, such as a solar panel, to make the station self-sufficient, and LoRaWAN/LoRaLAN or mobile telemetry technology, such as iridium modem, which would allow the user to have access remotely to the data measured and the conditions of the monitoring station. Additionally, a non-conductive coating, such as Plasti Dip®, can be added to the metal strip to prevent from any short circuit issues that may occur in case of contact with the electronic components, thereby reducing the risk of fire incidents.

Further enhancements can also be achieved by incorporating a velocimetry sensor or flow meter into the station, or by installing a hydraulic control structure, such as a weir. In this context, the appropriate weir equation can be employed in conjunction with the water level values already measured by the monitoring station to estimate flow rate with reasonable accuracy.

Future investigations should focus on a more comprehensive study of the effects of biofouling in the intake tubing, as well as potential strategies to mitigate this fouling effect. Additionally, it is essential to explore the extent to which temperature compensation can be effectively achieved through laboratory studies conducted under extreme temperature conditions. These research avenues will enhance our understanding of the challenges associated with sensor accuracy and reliability in various environmental contexts.

All the components and sensors to build this device are commercially available. The transparent design and all the information provided here allows researchers and waterway managers to build and maintain the monitoring station themselves, which favours the monitoring of environmental parameters of various waterways around the world, including natural and artificial systems.

## CRediT authorship contribution statement

**Paulo V.R.M. Silva:** Conceptualization, Data curation, Formal analysis, Methodology, Software, Validation, Visualization, Writing – original draft, Writing – review & editing. **Robert B. James:** Writing – review & editing, Validation, Software, Methodology, Conceptualization. **Kathryn L. Russell:** Writing – review & editing, Supervision, Project administration, Methodology, Conceptualization. **Tim D. Fletcher:** Writing – review & editing, Supervision, Software, Methodology, Conceptualization. **Maria F.S. Gisi:** Writing – review & editing. **Oldrich Navratil:** Writing – review & editing, Visualization, Supervision. **Frederic Cherqui:** Writing – review & editing, Visualization, Supervision. **Etienne Cossart:** Writing – review & editing, Supervision.

## Declaration of competing interest

The authors declare that they have no known competing financial interests or personal relationships that could have appeared to influence the work reported in this paper.
