# Supplementary material for: An automated low-cost monitoring station for suspended sediments and water level
Source: HardwareX. 2024 Oct 18;20:e00594. doi: 10.1016/j.ohx.2024.e00594 (PMC11554638; doi:10.1016/j.ohx.2024.e00594)
Supplement: Supplementary data [file mmc1.docx]

**Supplementary Information: Assembly of the Automated Low-Cost Monitoring Station for Suspended Sediments and Water Level**

This document provides supplementary and detailed instructions on how to assemble this automated monitoring station.

1. **Electronics**

The connections between the MELT datalogger and the other components of the monitoring station (peristaltic pump, forward and reverse power relays, temperature sensors, water level sensor, 12V battery and turbidity sensor) can be seen in Figure 1, which illustrates the electronic circuit schematic for the measurements.


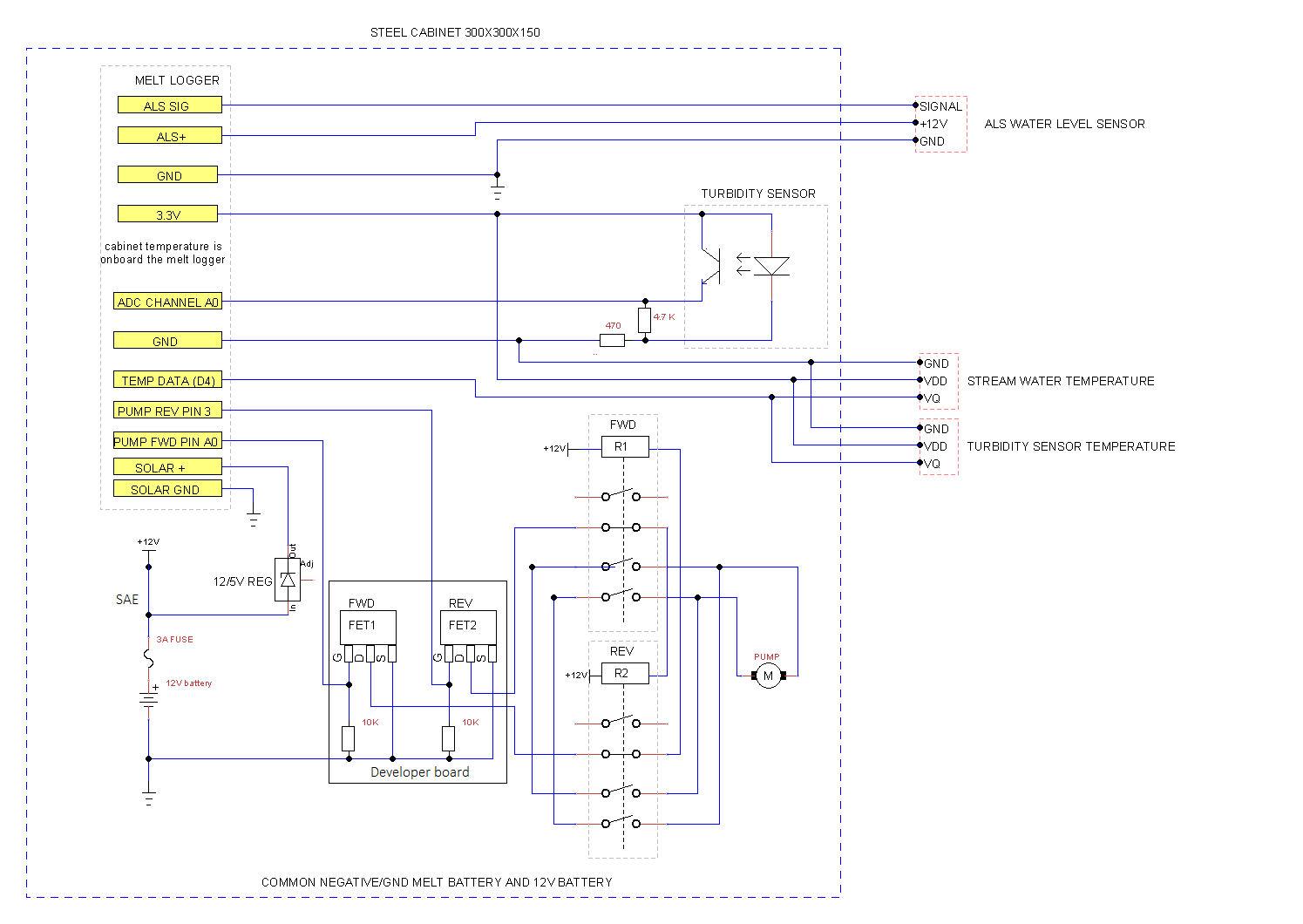


Figure 1 - Electronic circuit schematic for the measurements. Source: https://github.com/Robwerg/MobileTurbidity

1. **Preparation of the station cabinet:**

Begin by preparing the IP66-rated station cabinet (Figure 2). Drill the necessary holes for the installation of cable glands, the aluminum strip, and the aluminum rail (Figure 3).


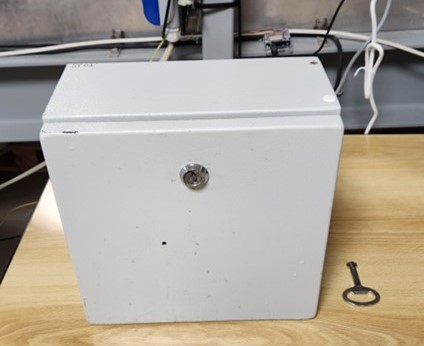


Figure 2 - IP66 cabinet.


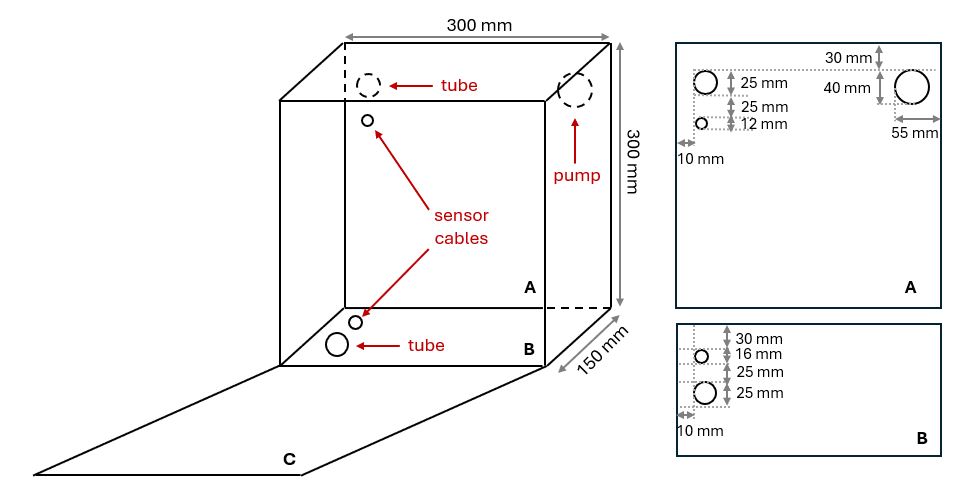


Figure 3 - IP66 cabinet preparation. “A” is the back wall of the cabinet. Three holes were made on this wall to pass the pump, the temperature sensor cable and the outlet tube. “B” is the base wall of the cabinet. Two holes were made on this wall to pass the inlet tube and the water pressure sensor cable. “C” is the lid of the cabinet.

An aluminium strip should be prepared and fixed to the cabinet to hold the battery securely inside. Additionally, an aluminium rail must be fixed to the back wall of the cabinet to hold both switches (power relays). The position and dimensions of both structures are shown in Figure 4. Both structures are fixed using metal screws. The material and width of both structures do not influence the performance of the equipment.


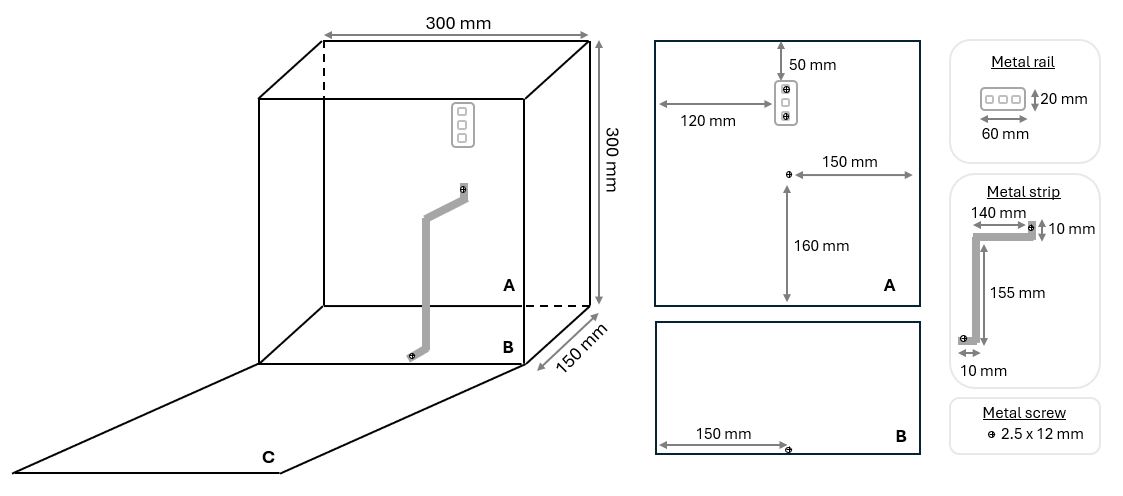


Figure 4 - Details of the cabinet preparation. "A" is the back wall of the cabinet, where the metal rail and one end of the strip are fixed. "B" is the bottom wall of the cabinet, where the other end of the metal strip should be fixed.

1. **Battery installation:**

Insert the 12V battery into the cabinet and secure it using the aluminum strip (Figure 5).


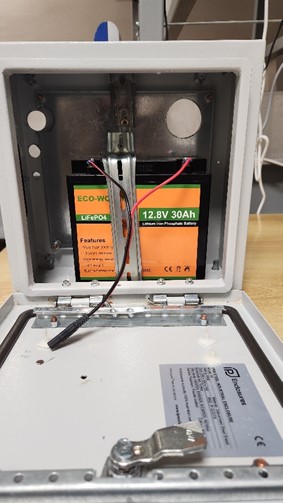


Figure 5 - Fixing the battery with the metal strip.

1. **Peristaltic pump installation:**

A sealing gasket (Figure 6A) was added to the pump to ensure that its assembly was sufficiently sealed, preventing water leakage into the monitoring station (Figure 6B). The gear motor should be put inside the cabinet by passing it through the hole (from the outside to the inside – Figure 6C), so the electrical part of the pump is protected from the weather. The next step is to fix the peristaltic pump to the external part of the cabinet with four metal screws (Figure 6D).


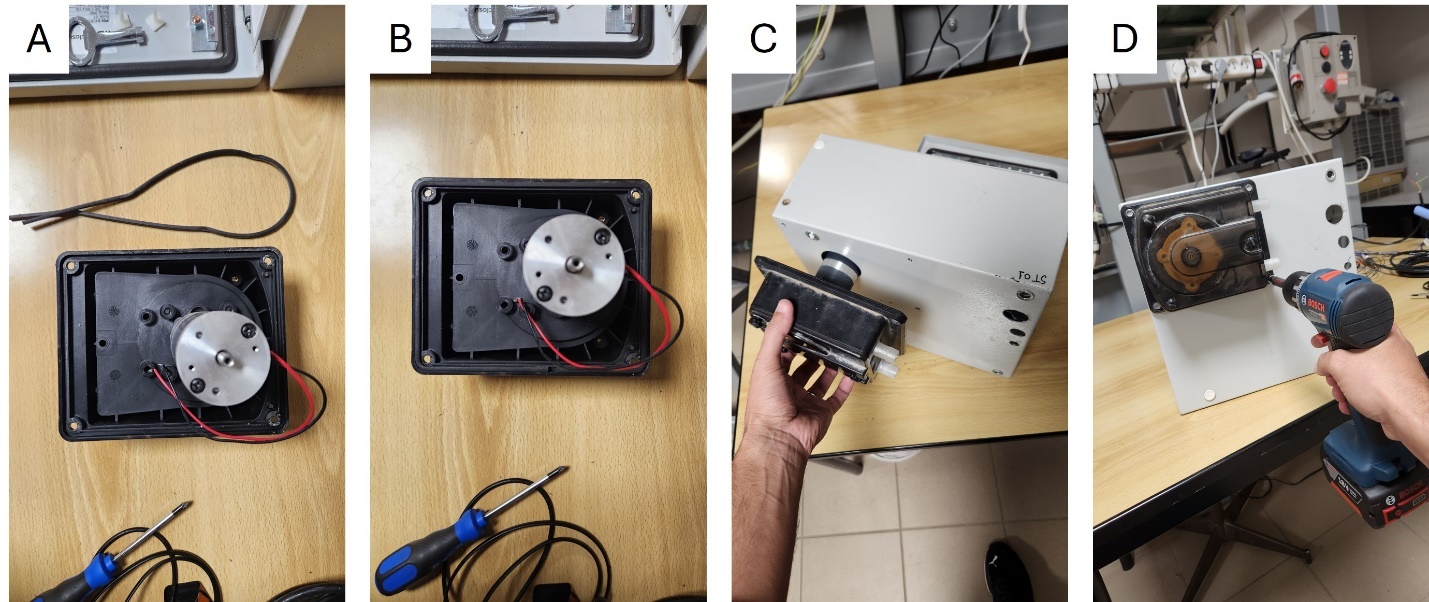


Figure 6 - Installation of the peristaltic pump. The pump was installed outside of the back wall of the cabinet by passing the gear motor through the hole and fixing its case with 4 metal screws - 2.5 x 12mm. A) The red arrow indicates the sealing gasket added to the pump to ensure that its assembly was sufficiently sealed. B) Shows the pump with the sealing gasket. C) Assembling the pump from the outside of the cabinet. D) The red arrows indicate where the pump is secured by the screws.

1. **Cable glands:**

Install the four cable glands to the cabinet, two cable glands of 25mm for the sampling tubes, one of 12mm for the cable of the temperature sensor, and one of 16mm for the cable of the water pressure sensor (Figure 7).


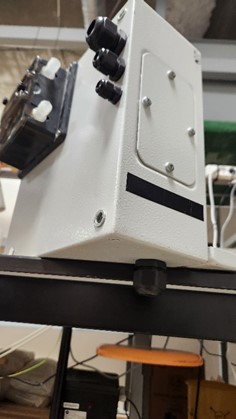

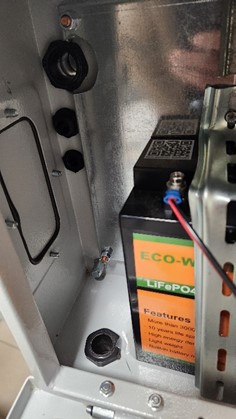


Figure 7 – Adding the cable glands.

1. **PCB mounting:**

Apply the PCB mount adhesives to the interior surface of the cabinet lid, and securely attach the PCB (Figure 8).


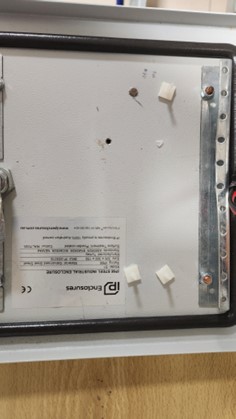

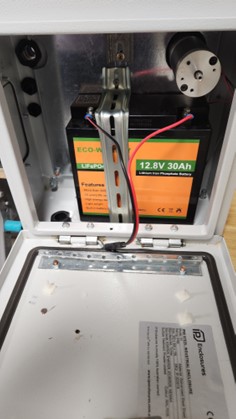

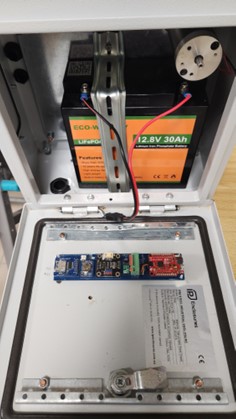


Figure 8 - PCB mounts adhesives installation and fixing the PCB.

1. **Water level sensor:**

Install the water level sensor by passing its cable from the outside of the cabinet to the inside (Figure 9).


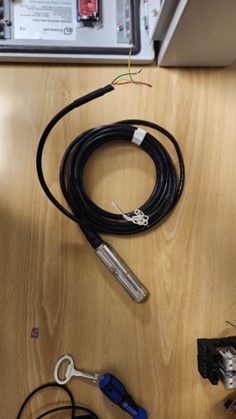

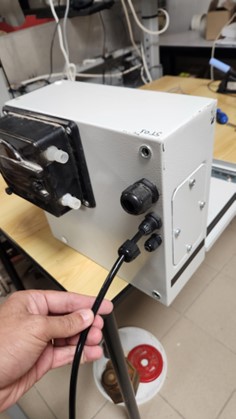

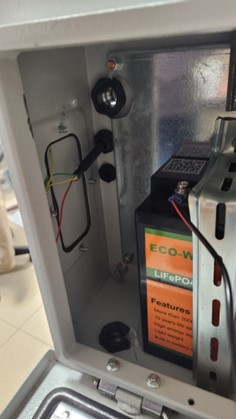


Figure 9 - Assembling the water level sensor.

1. **Temperature sensor:**

Install the temperature sensor by passing its cable from the outside of the cabinet to the inside (Figure 10).


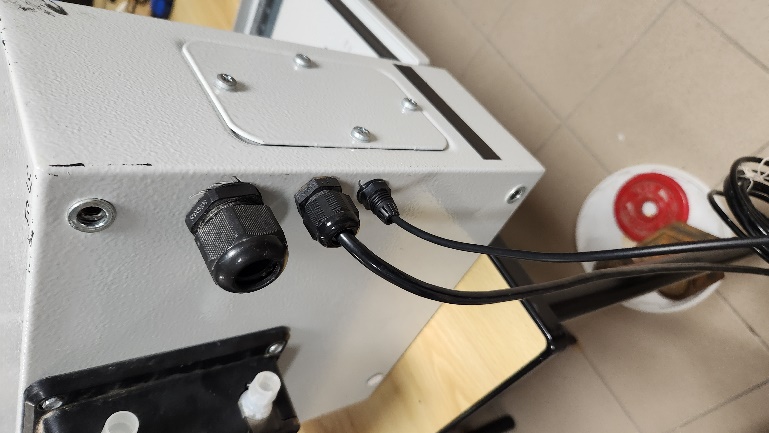

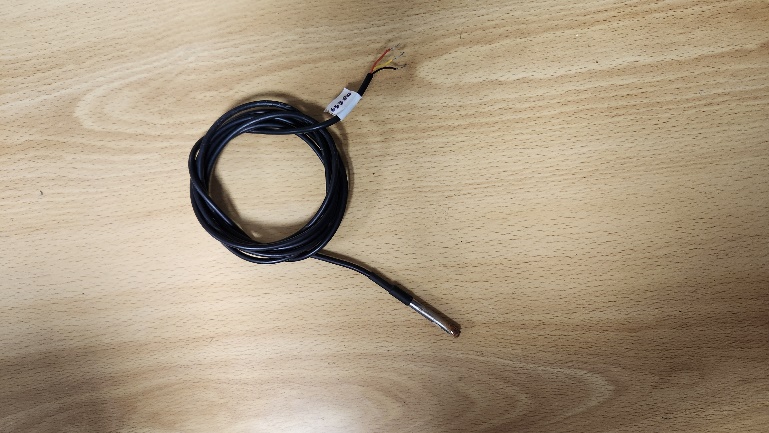

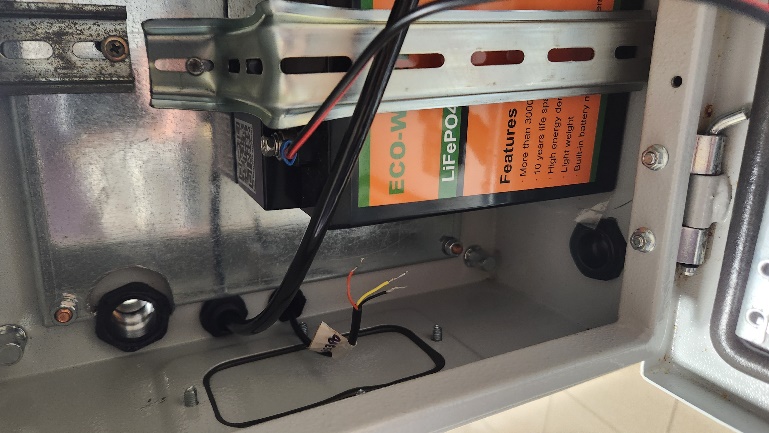


Figure 10 - Assembling the temperature sensor.

1. **Installation of the temperature sensor within the turbidity sensor enclosure**

The 1-wire temperature sensor (DS18B20) is installed using a hole (with a size of the temperature sensor diameter) on the top plastic of the turbidity sensor (black part), and then passing the wire temperature sensor through it. After this process, glue is added to the outside to better fix the wires and make the connection more robust (Figure 11)


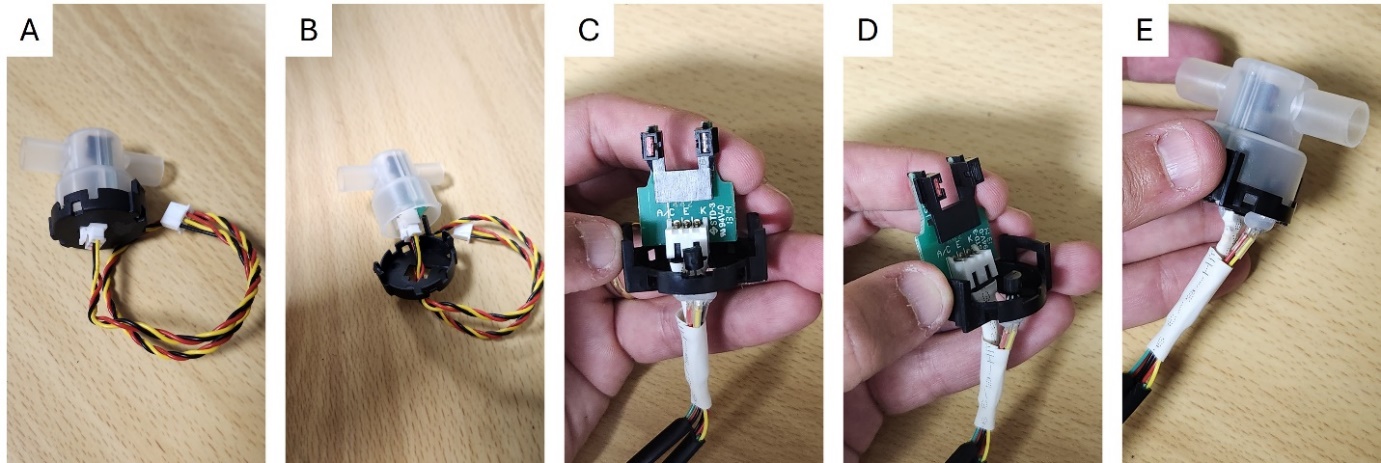


Figure 11 - Assembling the temperature sensor within the enclosure of the turbidity sensor. A and B) Turbidity sensor before adding the temperature sensor. Red arrows indicate where the hole should be done. C and D) The red arrows point to the temperature sensor that has been added inside the turbidity sensor housing. E) Turbidity sensor ready to be installed into the cabinet.

1. **Voltage converter preparation:**

Prepare the voltage converter by installing a fuse (Figure 12), as shown in Figure 1.


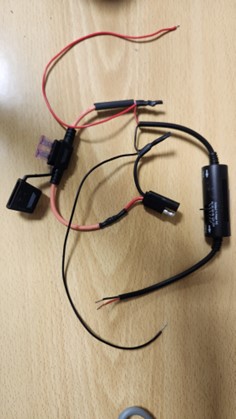

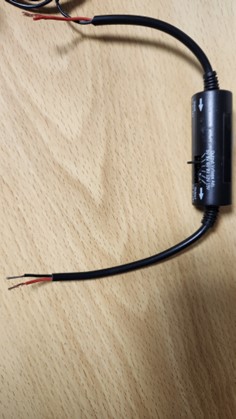


Figure 12 - Adding the fuse to the system to protect it. Preparing the voltage converter.

1. **Power relays:**

Begin wiring the power relays (Figure 13) in accordance with the provided electronic circuit schematic (Figure 1).


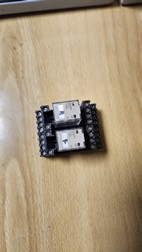

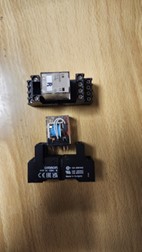

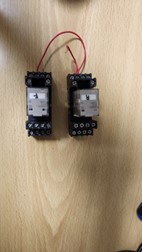

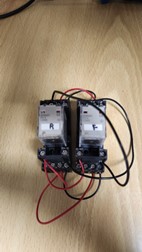

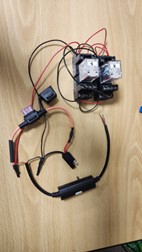


Figure 13 - Wiring power relays and adding a fuse.

1. **Developer board and PCB connections:**

Proceed with connecting the developer board, the pump, the voltage converter, and the sensors to the PCB and power relays (Figure 14), as shown in Figure 1. After that, mount the relays onto the aluminium rail (Figure 15).


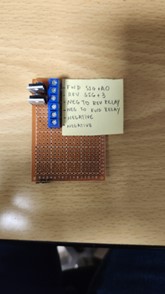

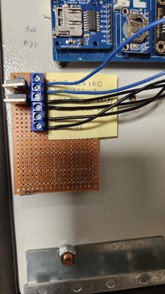

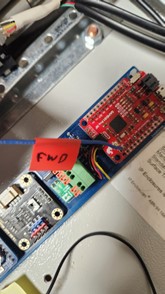

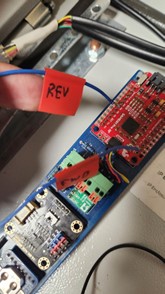


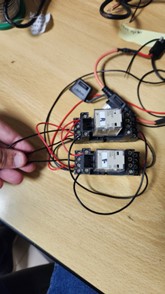

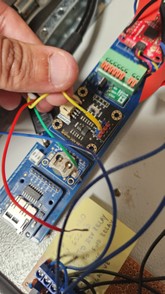

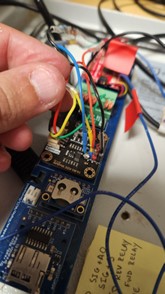

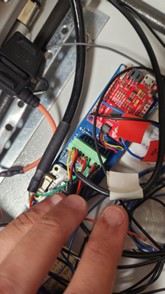


Figure 14 - Wiring all the components together.


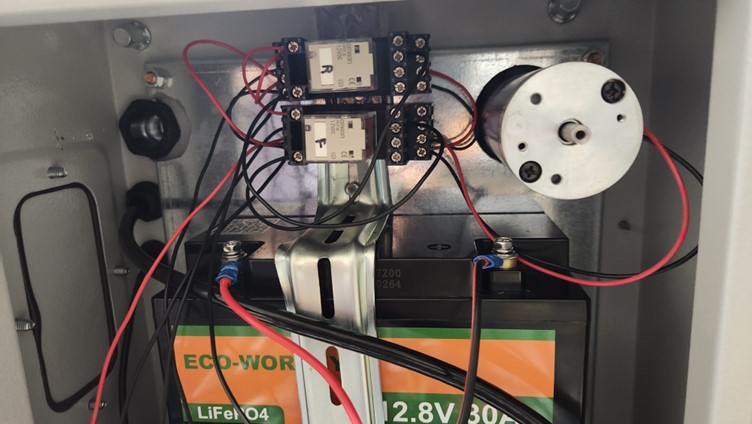


Figure 15 - Mounting the power relays after doing all connections onto the aluminium rail.

1. **SD card:**

Insert the SD card into the appropriate slot (Figure 16).


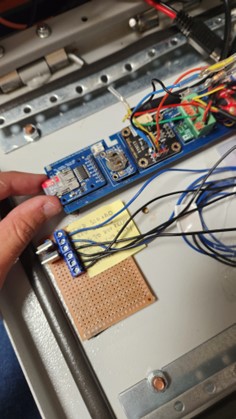

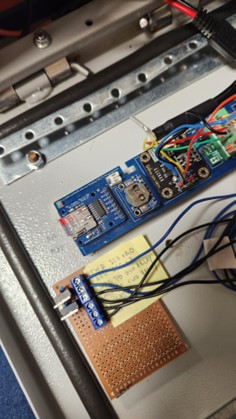


Figure 16 - Adding the SD card.

1. **Final connections:**

Install the intake tube and turbidity sensor (Figure 17). Connect the 12V battery to the PCB (Figure 18).


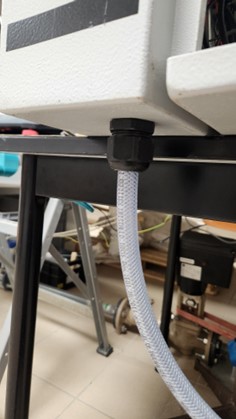

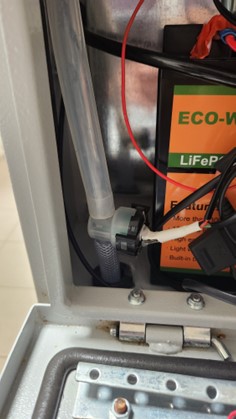

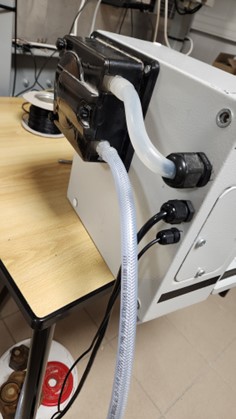


Figure 17 - Assembling the intake system (tubes and turbidity sensor).


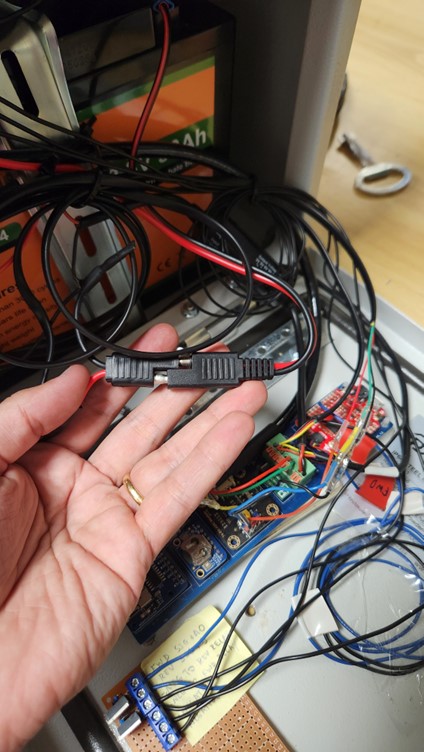


Figure 18 - Connecting the battery to the PCB.

Finally, Figure 19 provides a visual overview of the fully assembled monitoring station.


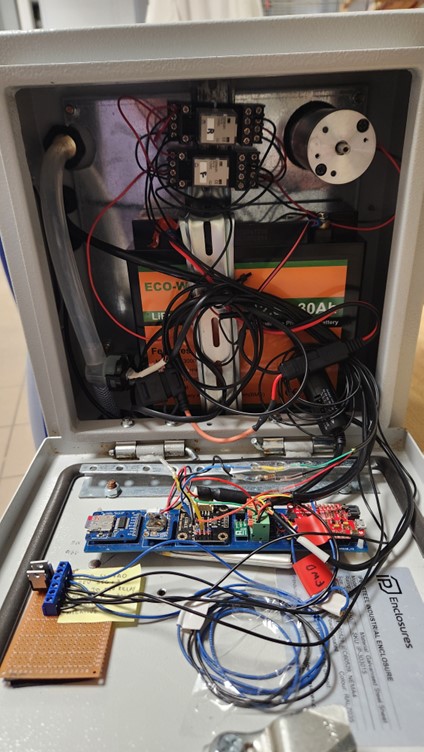


Figure 19 - Visual overview of the fully assembled monitoring station.
